# Long-term regional trends of nitrogen and sulfur deposition in the United States from 2002 to 2017

**DOI:** 10.5194/acp-22-12749-2022

**Published:** 2022-09-30

**Authors:** Sarah E. Benish, Jesse O. Bash, Kristen M. Foley, K. Wyat Appel, Christian Hogrefe, Robert Gilliam, George Pouliot

**Affiliations:** 1 US Environmental Protection Agency, Research Triangle Park, NC 27711, USA

## Abstract

Atmospheric deposition of nitrogen (N) and sulfur (S) compounds from human activity has greatly declined in the United States (US) over the past several decades in response to emission controls set by the Clean Air Act. While many observational studies have investigated spatial and temporal trends of atmospheric deposition, modeling assessments can provide useful information over areas with sparse measurements, although they usually have larger horizontal resolutions and are limited by input data availability. In this analysis, we evaluate wet, dry, and total N and S deposition from multiyear simulations within the contiguous US (CONUS). Community Multiscale Air Quality (CMAQ) model estimates from the EPA’s (Environmental Protection Agency) Air QUAlity TimE Series (EQUATES) project contain important model updates to atmospheric deposition algorithms compared to previous model data, including the new Surface Tiled Aerosol and Gaseous Exchange (STAGE) bidirectional deposition model which contains land-use-specific resistance parameterization and land-use-specific deposition estimates needed to estimate the differential impacts of N deposition to different land use types. First, we evaluate model estimates of wet deposition and ambient concentrations, finding underestimates of SO_4_, NO_3_, and NH_4_ wet deposition compared to National Atmospheric Deposition Program observations and underestimates of NH_4_ and SO_4_ and overestimates of SO_2_ and TNO_3_ (HNO_3_+NO_3_) compared to the Clean Air Status and Trends Network (CASTNET) ambient concentrations. Second, a measurement–model fusion approach employing a precipitation and bias correction to wet-deposition estimates is found to reduce model bias and improve correlations compared to the unadjusted model values. Model agreement of wet deposition is poor over parts of the West and Northern Rockies, due to errors in precipitation estimates caused by complex terrain and uncertainty in emissions at the relatively coarse 12 km grid resolution used in this study. Next, we assess modeled N and S deposition trends across climatologically consistent regions in the CONUS. Total deposition of N and S in the eastern US is larger than the western US with a steeper decreasing trend from 2002–2017; i.e., total N declined at a rate of approximately −0.30 kg N ha^−1^ yr^−1^ in the Northeast and Southeast and by −0.02 kg N ha^−1^ yr^−1^ in the Northwest and Southwest. Widespread increases in reduced N deposition across the Upper Midwest, Northern Rockies, and West indicate evolving atmospheric composition due to increased precipitation amounts over some areas, growing agricultural emissions, and regional NO_*x*_/SO_*x*_ emission reductions shifting gas–aerosol partitioning; these increases in reduced N deposition are generally masked by the larger decreasing oxidized N trend. We find larger average declining trends of total N and S deposition between 2002–2009 than 2010–2017, suggesting a slowdown of the rate of decline likely in response to smaller emission reductions. Finally, we document changes in the modeled total N and S deposition budgets. The average annual total N deposition budget over the CONUS decreases from 7.8 in 2002 to 6.3 kg N ha^−1^ yr^−1^ in 2017 due to declines in oxidized N deposition from NO_*x*_ emission controls. Across the CONUS during the 2002–2017 time period, the average contribution of dry deposition to the total N deposition budget drops from 60 % to 52 %, whereas wet deposition dominates the S budget rising from 45 % to 68 %. Our analysis extends upon the literature documenting the growing contribution of reduced N to the total deposition budget, particularly in the Upper Midwest and Northern Rockies, and documents a slowdown of the declining oxidized N deposition trend, which may have consequences on vegetation diversity and productivity.

## 1 Introduction

Human activity doubled the amount of reactive nitrogen (*N*_r_) in the environmental globally over the past century (Fowler et al., 2013). Major sources responsible for the increase include fossil fuel combustion from vehicles and electric utilities emitting nitrogen oxides (NO_*x*_ =NO + NO_2_) and sulfur dioxide (SO_2_), as well as agricultural activities releasing ammonia (NH_3_) (Galloway et al., 2008). After entering the atmosphere, the major nitrogen (N) and sulfur (S) removal pathways occur by precipitation (wet deposition) or uptake by surfaces, such as terrestrial and aquatic vegetation (dry deposition). Consequences of deposition include risks to public health (Townsend et al., 2003) and damages to ecosystems, such as acidification of soil and waterways, changes to species composition (Bobbink et al., 2010), and massive cyanobacteria and algal blooms (Jaworski, 1990), which may cause physiological stress and compromise immune function among wildlife (Refsnider et al., 2021). Despite adverse effects of deposition to humans and the environment, the total (wet + dry) N and S budgets remain uncertain, particularly for sensitive ecosystems in critical regions due to observational gaps.

Deposition monitoring and assessment played a decisive role informing the United States (US) Clean Air Act Amendments (CAAA) of 1990 (United States Congress, 1990). Wet deposition, sampled weekly in rain or snow, has been measured by the National Atmospheric Deposition Program (NADP) National Trends Network (NTN) since 1978. Dry-deposition measurements are inferred by combining weekly measured concentrations from the Clean Air Status and Trends Network (CASTNET) and modeled deposition velocities from the multilayer model (MLM) (Meyers et al., 1998). Dry-deposition modeling is still uncertain, particularly for land use and dry-deposition schemes in models and emission data. Despite providing critical deposition information, the limited number of NADP and CASTNET sites in essential locations, such as areas with complex terrain, near urban centers, at high elevation, or in forest ecosystems, restrict a thorough understanding on the amount and consequences of deposition. For instance, strong concentration gradients in N deposition have been documented over urban areas such as Boston, Massachusetts (Rao et al., 2013), and near Baltimore, Maryland (Bettez et al., 2013), and along coastlines like the Chesapeake Bay (Loughner et al., 2016). Additionally, networks are unable to capture the full budget of *N*_r_. For instance, organic N species are estimated to contribute ~ 25 % to total *N*_r_ (Jickells et al., 2013) but are not easily quantified by the NTN due to limitations in field and laboratory techniques (Walker et al., 2012).

Chemical transport models (CTMs) can be used to study deposition relationships and trends for locations without measurements and compounds that are challenging to quantify (e.g., organic N). However, to provide reliable estimates, CTMs estimates must first be evaluated with measurement data. Various studies have compared N and S wet deposition and concentrations estimated by the Community Multiscale Air Quality (CMAQ) model (https://www.epa.gov/cmaq, last access: 11 March 2022) with observed values in the US (Xing et al., 2015; Zhang et al., 2018; Appel et al., 2011), providing a wide range of agreement with measurements. Zhang et al. (2018) found adjusting CMAQv5.0.2 wet-deposition estimates with observed precipitation results in a larger negative normalized mean bias (NMB) than the original CMAQ estimates for TNO_3_ (NO_3_+HNO_3_, −31.6% unadjusted vs. −35.6% adjusted), NH_*x*_ (NH_3_+NH_4_, −30.9% unadjusted vs. −35.1% adjusted), and total S (−5.1% unadjusted vs. −10.5% adjusted). Similar model agreement was published using CMAQv4.7 (Appel et al., 2011) and attributed to the coarse grid resolution (36 km). In Europe, Theobald et al. (2019) characterized the model bias of wet deposition of NO_*x*_, NH_*x*_, and SO_*x*_ from six CTMs between 1990 and 2010 to range from 30 %–40 %. Biased model simulations may lead to incorrect scientific conclusions, such as critical-load designations (Williams et al., 2017). Methods to fuse model estimates with measured values can therefore be used to provide more reliable insights into “biogeochemical cycles and assessments of ecosystems and human health effects” (World Meteorological Organization, 2017). As such, the NADP Total Deposition Science Committee (TDep; see https://nadp.slh.wisc.edu/committees/tdep/, last access: 11 March 2022) advances methods to improve estimates of atmospheric deposition from CMAQ (see Schwede and Lear, 2014). TDep products only employ a fusion approach to dry deposition and currently use an older version of CMAQ, although efforts to update the model version and incorporate wet-deposition fusion described herein are ongoing.

In this study, we assess deposition from the EPA’s (Environmental Protection Agency) Air QUAlity TimE Series (EQUATES) project (https://www.epa.gov/cmaq/equates, last access: 11 March 2022), which utilized the CMAQ version 5.3.2 modeling platform. The EQUATES project used consistent methodology (e.g., input data, processing methods, model versions) with deposition science updates compared to previous model versions, including the new Surface Tiled Aerosol and Gaseous Exchange (STAGE) bidirectional deposition model and improvements to organic N chemistry. Long-term air quality simulations like EQUATES are used by the scientific community in ecological and epidemiological assessments, including critical-load analyses. By employing these science updates in the model, consistent methodology, finer grid spacing (12 km), and a bias adjustment to wet deposition, we evaluate our best understanding of simulated deposition trends in the US. We evaluate the model agreement with observations of wet deposition and concentrations used to infer dry deposition and describe a measurement–model fusion technique in Sect. 2. In Sect. 3, we focus on the modeled trend and budget of N and S deposition over nine climatologically consistent regions within the contiguous US (CONUS) over a period of large emission reductions designated by the CAAA. Section 4 ends with Conclusions.

## 2 Methods and materials

### 2.1 EQUATES model configuration

The long-term EQUATES simulations from 2002–2017 were run using the CMAQ model version 5.3.2. Emissions were from different national emission inventories (NEIs) or used a base year NEI and estimated emissions for other years by creating scaling factors based on activity data and emission control information (Xing et al., 2013; Foley et al., 2020). A summary of recent CMAQ version 5.3 model updates and their impact on modeled concentrations is provided in Appel et al. (2021). The Weather Research and Forecasting version 4.1.1 (WRFv4.1.1) model was used for meteorology. Lateral boundary conditions for the 12 km grid spacing CONUS domain used in this study were provided by a 108 km grid spacing Northern Hemispheric simulation. Hemispheric and North American emission inventories were specifically prepared for EQUATES to ensure consistent input data and methods across all years. The Surface Tiled Aerosol and Gaseous Deposition (STAGE) option in CMAQv5.3.2 (Galmarini et al., 2021) was used to estimate atmospheric dry-deposition rates. The STAGE dry-deposition option generally performs similar to M3Dry (the other dry-deposition option in CMAQv5.3.2) when compared to network observations of ambient gaseous and aerosol pollutant concentrations (Appel et al., 2021) while providing additional land-use-specific deposition data useful for assessments of ecosystem exposure (Hood et al., 2021).

### 2.2 US deposition observations

**Figure 1.**
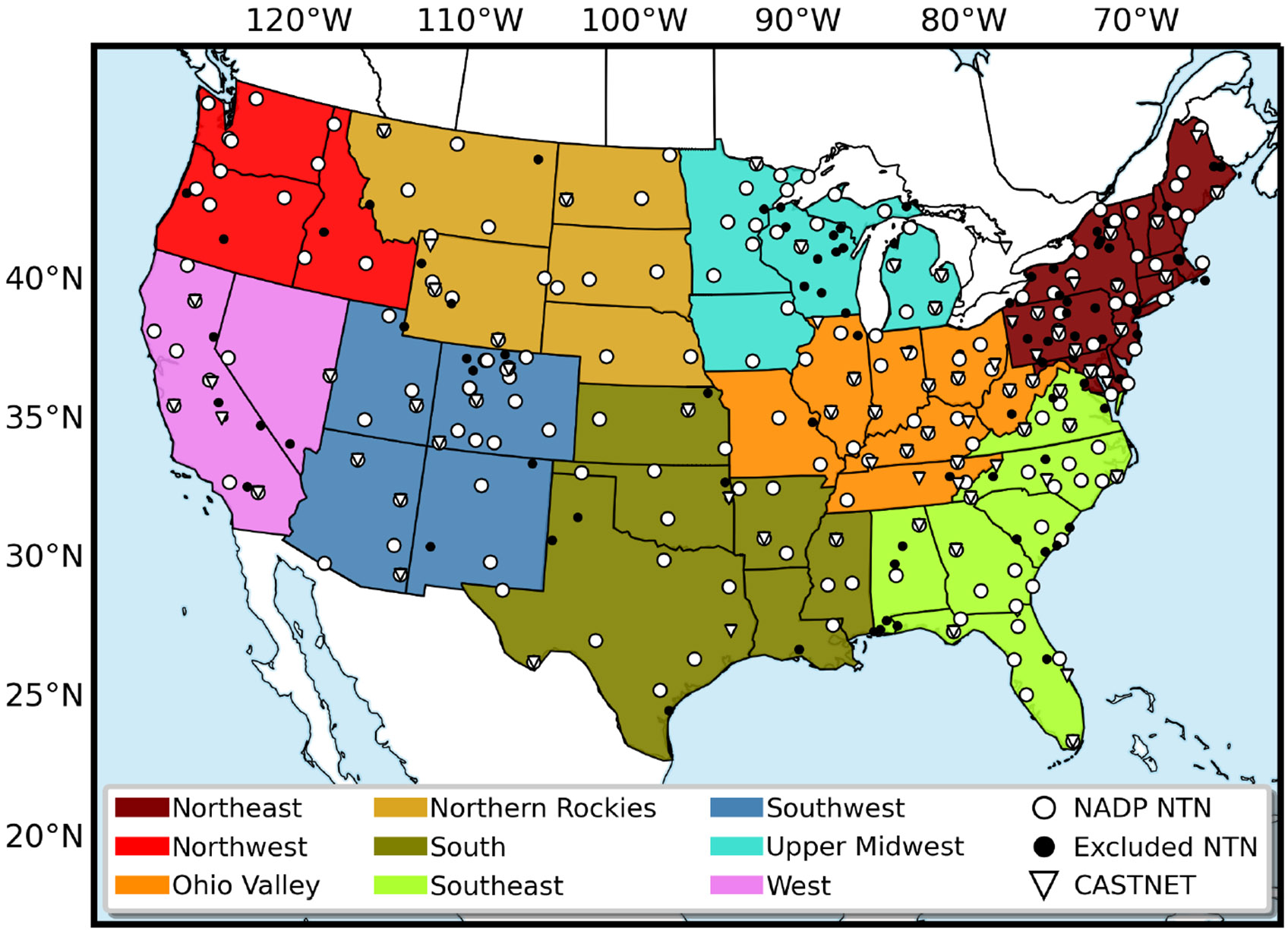
Locations of the 200 National Atmospheric Deposition Program (NADP) National Trends Network (NTN, white circles) and 75 Clean Air Status and Trends Network (CASTNET, triangles) sites examined in this study. NADP NTN sites shown in black circles did not meet completeness criteria thresholds and therefore were not included in this analysis. Color-coded US climate regions shown in this map are referred to throughout this analysis. The black-bordered white circles indicate NADP NTN sites that meet annual completeness criteria for 13 years of the time series and are examined in the model evaluation presented in Sect. 3.1.

We assess CMAQ’s ability to reproduce annual accumulated wet deposition and ambient concentrations of N and S species measured by the NADP NTN and EPA’s CASTNET networks (Fig. 1). Since anthropogenic sources dominate deposition and their area of influence is largely regional (Paulot et al., 2013), we evaluate the agreement over CONUS climate regions from 2002–2017 following the NOAA definition defined by Karl and Koss (1984), although these groupings do not imply the regions are isolated from each other. The nine climate regions include the Northwest, West, Northern Rockies, Southwest, South, Ohio Valley, Southeast, and Northeast as shown in Fig. 1.

The NADP’s NTN (http://nadp.slh.wisc.edu, last access: 11 March 2022) currently collects weekly precipitation samples at 263 locations across the US. Samples are analyzed by ion chromatography or flow injection analysis to quantify wet deposition of nitrate (NO_3_), ammonium (NH_4_), and sulfate (SO_4_), as well as several other compounds. First, modeled wet deposition of NO_3_, NH_4_, and SO_4_ is calculated using the approach by Appel et al. (2011) that accounts for chemical transformations of several species in the aqueous phase. Then, the model and observations are paired in time and space and annually accumulated. Following similar completeness criteria thresholds as in Zhang et al. (2019), NTN sites must be available for at least 13 of the 16 years simulated and have at least 60 % annual coverage each year to be considered for analysis. Applying these criteria, 200 NTN sites meet the minimum requirements for our analysis (Table S1 in the Supplement). The CASTNET measures concentrations and estimates dry deposition at 99 sites in the US (https://www.epa.gov/castnet, last access: 11 March 2022). Weekly ambient concentrations of gases and particles are collected with an open-face three-stage filter pack. Since the reported dry-deposition values estimated by CASTNET are model derived, we instead evaluate the CMAQ performance simulating the ambient air concentrations of sulfur dioxide (SO_2_), sulfate (SO_4_), total nitrate (TNO_3_ =NO_3_+HNO_3_), and ammonium (NH_4_). CASTNET sites available for at least 13 of the 16 years simulated with 75 % annual coverage each year are included in this analysis, resulting in 75 valid sites meeting the minimum requirements (Table S2). The EQUATES simulations are evaluated by comparison with available monitoring data using several model performance statistics (Simon et al., 2012).

### 2.3 Measurement–model fusion technique

The modeled wet-deposition fields are adjusted to account for input biases and uncertainty in the chemical and physical processes governing deposition. No corrections are applied to dry deposition due to limited dry-deposition measurements. Since model performance is improved for annual instead of seasonal values (Table S3; refer to Zhang et al., 2019, for detailed seasonal model evaluation), we apply a measurement–model fusion technique previously described by Zhang et al. (2019) to adjust the modeled annual wet-deposition fields of inorganic N (NO_3_+NH_4_) and S, briefly described here. First, the modeled wet-deposition fields from EQUATES are adjusted using observation-based 4 km precipitation fields generated by the Parameter-elevation Regressions on Independent Slopes Model (PRISM, https://prism.oregonstate.edu/, last access: 11 March 2022), annually accumulated and regridded to the 12 km CMAQ domain (${\text{EQUATES}}_{\text{precip-adj}}$). While wet deposition relies on the season and precipitation rate (Schichtel et al., 2019), the relationship between precipitation and wet deposition is more linear when annually accumulated. Since PRISM incorporates observed elevation and climatic factors to estimate precipitation, NTN and TDep maps also used these data to improve estimates of wet deposition over complex terrain (Schwede and Lear, 2014). In each grid cell, the EQUATES modeled annual total wet deposition (${\text{WD}}_{\text{mod}}$) is adjusted by the ratio of annually accumulated precipitation from PRISM (${\text{Precip}}_{\text{obs}}$) to the WRF estimated annual accumulated rainfall (${\text{Precip}}_{\text{mod}}$) (Eq. 1):

(1) $${\text{EQUATES}}_{\text{precip-adj}}=\frac{{\text{Precip}}_{\text{obs}}}{{\text{Precip}}_{\text{mod}}}\times {\text{WD}}_{\text{mod}}.$$

After adjusting simulated wet deposition by precipitation, an additional bias adjustment (${\text{EQUATES}}_{\text{bias-adj}}$) is applied using all NTN observations that meet annual data completeness, which varies year to year and includes sites of all classifications. For each NTN site, the median ratio between the ${\text{EQUATES}}_{\text{precip-adj}}$ wet deposition and the NTN observations is calculated for all sites within a 300 km radius. Then, the median biases are interpolated using universal kriging with a linear trend in the spatial coordinates and an exponential covariance structure using Python’s PyKrige module (https://github.com/GeoStat-Framework/PyKrige, last access: 11 March 2022). A cross-validation analysis employing this measurement–model fusion technique with CMAQv5.0.2 is presented in Zhang et al. (2019). The final wet-deposition bias adjustment (Eq. 2) is computed by multiplying the precipitation-adjusted wet deposition by the inverse of the bias field, b:

(2) $${\text{EQUATES}}_{\text{bias-adj}}=\frac{1}{b}\times {\text{EQUATES}}_{\text{precip-adj}}.$$

## 3 Results and discussion

### 3.1 Model performance

**Figure 2.**
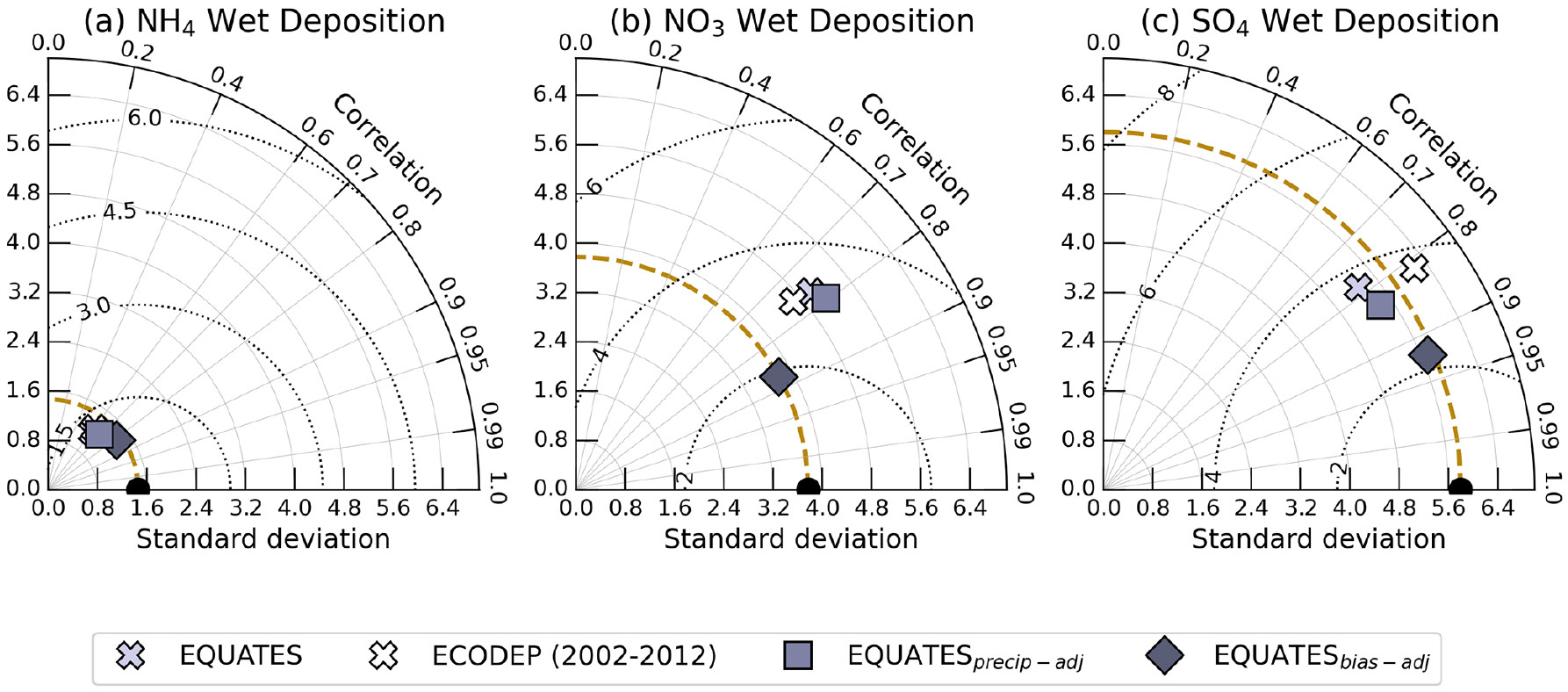
Taylor plot comparing annual accumulated wet deposition (kg ha^−1^) of NH_4_ (**a**), NO_3_ (**b**), and SO_4_ (**c**) collected at NTN sites (black circles, along the *x* axis) with model output. The symbols differentiate between the various models (EQUATES and ECODEP) and wet-deposition corrections as described in the text. The azimuthal angle denotes the Pearson correlation coefficient (*r*^2^); the gold dashed radial distance shows the standard deviation (kg ha^−1^); and the dotted semicircles centered at the observation marker (black circle) denote the root-mean-square error.

In this section, we investigate the EQUATES model performance, estimating wet deposition and ambient concentrations of N and S compounds throughout the US. The impact of the precipitation and bias correction technique on reproducing 2002–2017 accumulated measured wet deposition of NH_4_, NO_3_, and SO_4_ is shown in the Taylor diagram in Fig. 2 and summarized in Table 1. In addition, since previous modeling studies demonstrated moderate skill simulating wet deposition (Appel et al., 2011; Zhang et al., 2018), we also compare to an earlier time series utilizing CMAQv5.0.2 (ECODEP; 2002–2012 only). Little difference was found between EQUATES- and ECODEP-simulated wet-deposition statistics over the CONUS, although the improvement in the EQUATES estimates is more noticeable over regions with improved precipitation estimates. For example, a high precipitation bias in the WRFv3.4 meteorology used for ECODEP is reduced or eliminated in the WRFv4.1.1 meteorology used for EQUATES. The decrease in bias is likely attributed to the implementation of a hybrid coordinate system in WRFv4.1.1 which has been found to improve precipitation estimates at high-elevation sites in the western US (Beck et al., 2020), although the EQUATES precipitation is still biased low on average relative to PRISM, particularly in the Northwest (Fig. S1 in the Supplement). The close grouping of markers in Fig. 2a compared to the other species presented in Fig. 2 reflect the smaller standard deviation in the NH_4_ wet-deposition measurements (1.5 kg ha^−1^), which are comparable and slightly underestimated among the various models and adjustments. Applying the precipitation adjustment (${\text{EQUATES}}_{\text{precip-adj}}$) reduces the underestimation of NH_4_ and SO_4_ wet deposition and increases the overestimation of NO_3_ wet deposition compared to the unadjusted model output (Table 1). The bias-adjusted NH_4_, NO_3_, and SO_4_ wet-deposition estimates have a similar standard deviation as the observations (falling near the dashed arc) and also feature a lower root-mean-square error (RMSE) (dotted semicircles) and higher correlation compared to the other estimates. Interannually, the bias correction to EQUATES output reduces the NMB of NH_4_ and SO_4_ wet deposition by 10 %–30 % and 5 %–20 %, respectively, compared to the unadjusted model output, with the largest improvement occurring from 2010–2017 (Fig. S2). Corrections applied to modeled NO_3_ wet deposition reduce the NMB by ~ 20 % in the beginning of the time series (2002–2009) and < 10% at the end (2010–2017), possibly related to more uncertainty in emissions earlier in the earlier time period. The bias corrections to NH_4_ wet deposition are particularly noteworthy in the Upper Midwest, Northern Rockies, Ohio Valley, and South (Fig. S3), improving the agreement between the modeled and observed standard deviation, increasing the correlation, and lowering the RMSE compared to the unadjusted model values. The bias adjustment greatly improves the agreement of NO_3_ and SO_4_ wet deposition to observations in the Northeast, Northwest, and Ohio Valley by similarly improving the model performance metrics (Figs. S4 and S5). Due to the large improvements in estimating wet deposition using the measurement–model fusion approach, we use these corrections when assessing wet and total deposition throughout the rest of the analysis.

**Figure 3.**
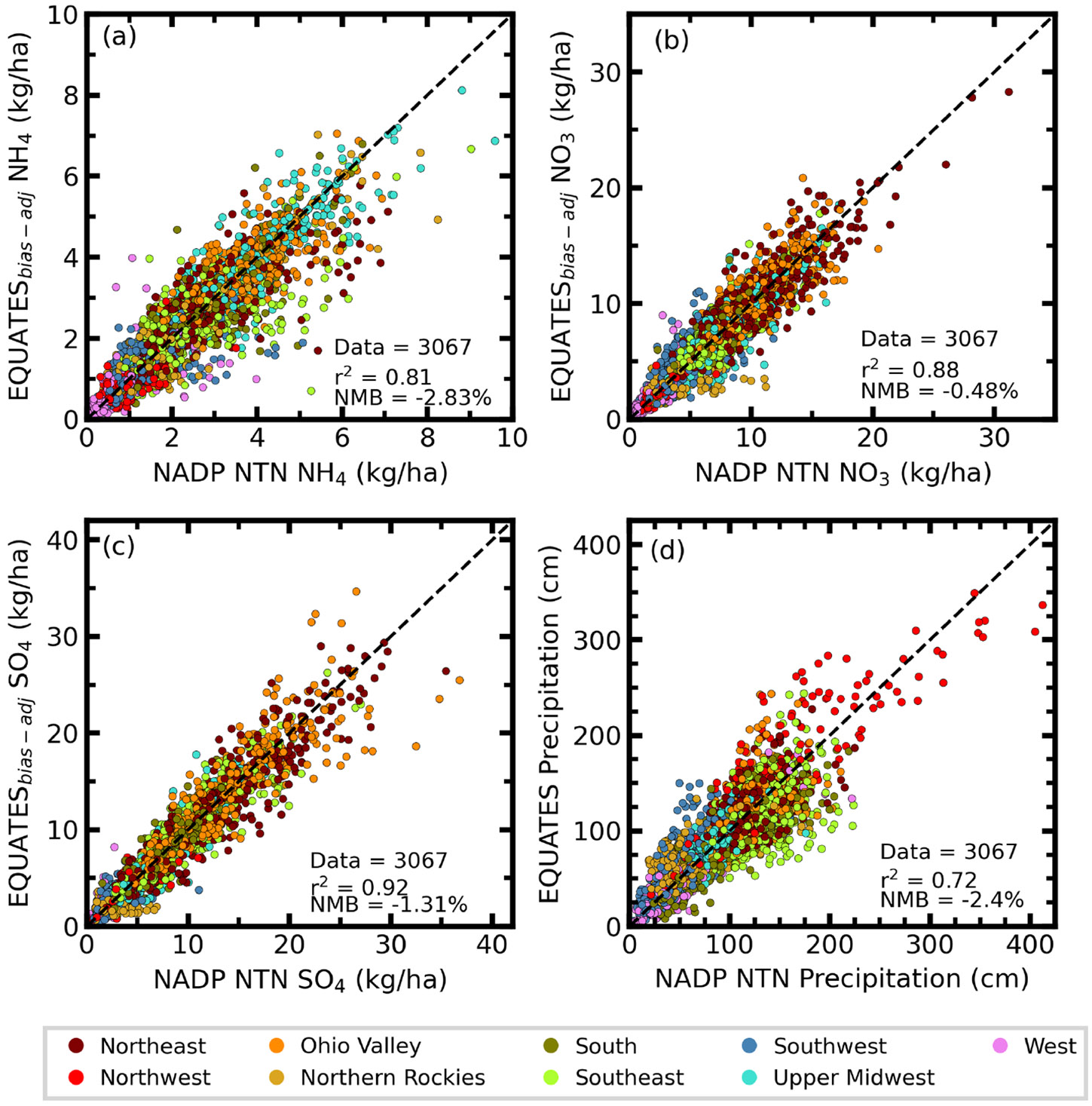
Scatterplots of annual accumulated bias-adjusted modeled and NTN observed wet deposition (kg ha^−1^) of ammonium (**a**, NH_4_), nitrate (**b**, NO_3_), and sulfate (**c**, SO_4_) from 2002 to 2017 colored by the climate region. Panel (**d**) shows NADP NTN observed and modeled precipitation (cm).

Figure 3 provides an overall comparison between the bias-corrected EQUATES wet deposition and NTN observations, along with an evaluation of simulated and observed precipitation. Table 2 provides summary statistics of wet deposition and precipitation for each climate region. A slight negative bias of modeled precipitation and bias-corrected NH_4_, NO_3_, and SO_4_ wet deposition is found for the entire CONUS, although agreement across the climate regions varies widely. The model generally exhibits better performance in the eastern climate regions, especially the Ohio Valley and Upper Midwest, compared to the rest of the CONUS. Agreement in the West, Northwest, and Northern Rockies is particularly poor, likely due to the errors simulating precipitation in the complex terrain (Table 2). In these western climate regions, precipitation is generally overestimated, consistent with previous model versions (Zhang et al., 2018).

The 16-year annual accumulated NH_4_ wet deposition is highest in the Upper Midwest, seen in both the model and measurements, and lowest in the Northwest. The model generally underestimates NH_4_ wet deposition for all regions except the Ohio Valley, with MB values ranging from 0.03 kg ha^−1^ in the Ohio Valley to −0.15 kg ha^−1^ in the Southeast. The correlation coefficients for wet NH_4_ deposition are on average stronger in the northern (*r*^2^ > 0.66) than southern US (*r*^2^ < 0.60). The weaker correlation between the measured and modeled trends of NH_4_ wet deposition (*r*^2^ = 0.43) is impacted by the smaller magnitude of the observed and modeled NH_4_ trends and reflects moderate model performance reproducing decreasing trends in many parts of the southern US and increasing trends in the northern US (Fig. S6). The 16-year annual accumulated NO_3_ wet deposition is also highest in the Upper Midwest but lowest in the West. The simulation overestimates NO_3_ wet deposition in the West, South, Ohio Valley, and Southwest with MB values all approximately 0.10 kg ha^−1^, while all other regions are underestimated with a MB ranging from −0.01 in the Northwest to −0.39 kg ha^−1^ in the Northern Rockies. Correlation coefficients for NO_3_ wet deposition are greater than 0.60 in all regions except the Southwest and Northern Rockies (*r*^2^ ~ 0.50). Similar to NO_3_ wet deposition, the 16-year annual accumulated wet SO_4_ deposition is also highest in the Upper Midwest and lowest in the West. The simulation overestimates SO_4_ wet deposition across most of the southern US, where the MB ranges from 0.01 (Southwest) to 0.16 kg ha^−1^ (West). Most other regions show an underestimation of SO_4_ wet deposition with MB ranging from −0.03 (Ohio Valley) to −0.32 kg ha^−1^ (Northeast). Correlation coefficients are strongest for SO_4_ wet deposition than the other species, particularly in the northern US where *r*^2^ values are generally greater than 0.80. Like NH_4_ and NO_3_ wet deposition, correlation coefficients for SO_4_ wet deposition are weakest in the Southwest and West. The model also captures the clear downward trends of NO_3_ and SO_4_ wet deposition (*r*^2^ = 0.79 and 0.94, respectively), although the magnitudes of the decreasing modeled trends are slightly underestimated (Fig. S6).

**Figure 4.**
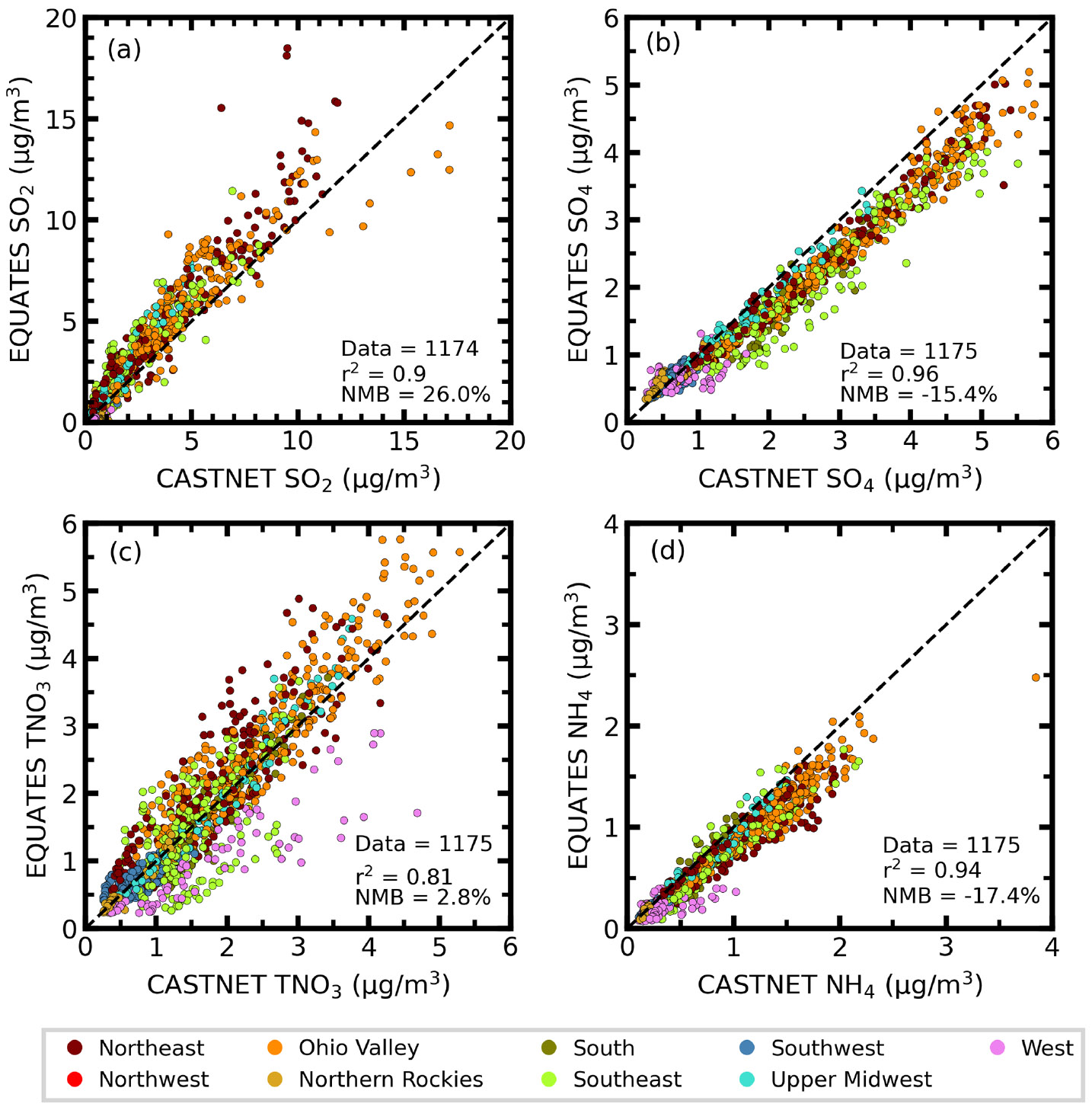
Scatterplots comparing 2002–2017 annual average concentrations (μg m^−3^) of sulfur dioxide (**a**, SO_2_), sulfate (**b**, SO_4_), total nitrate (**c**, TNO_3_), and ammonium (**d**, NH_4_) between EQUATES output and CASTNET observations (*N* = 1175). The marker color corresponds to the climate region. Data from CASTNET sites with at least 75 % valid data available for each of the 13 years analyzed are included in the analysis shown.

To indirectly evaluate the ability of the EQUATES simulations to reproduce dry deposition, we compare the modelsimulated annual average concentrations of SO_2_, SO_4_, TNO_3_, and NH_4_ with observations collected at CASTNET sites in Fig. 4 and in Table 3. Correlations between measured and modeled mean concentrations show good agreement for NH_4_ (*r*^2^ = 0.94), TNO_3_ (*r*^2^ = 0.81), SO_2_ (*r*^2^ = 0.90), and SO_4_ (*r*^2^ = 0.96). Overall, the model shows negatives biases for NH_4_ and SO_4_ concentrations (−17.4% and −15.4%, respectively) and positive biases for TNO_3_ and SO_2_ concentrations (2.8 % and 26.0 %, respectively) but generally improved from CMAQv5.0 (Xing et al., 2015). The highest 16-year average concentrations for all species are found in the Upper Midwest for both the simulations and observations. The model underestimates concentrations of NH_4_ across all regions, with MB ranging from −0.02 (Northern Rockies) to −0.22 μg m^−3^ (Ohio Valley). Correlation coefficients for NH_4_ concentrations are generally strong (*r*^2^ > 0.80) everywhere, except the West and Southwest (*r*^2^ = 0.09 and 0.39, respectively). Average modeled TNO_3_ concentrations are mostly overestimated in the northern US, with MB ranging from 0.02 (Northern Rockies) to 0.26 μg m^−3^ (Ohio Valley), and underestimated in the southern US, with MB ranging from −0.01 (Southwest) to −0.64μgm^−3^ (West). Concentrations of SO_2_ are generally overestimated except in the western regions (West, Southwest, and Northern Rockies), and strong correlations are found throughout all regions. The model underestimates SO_4_ concentrations in most regions, with MB ranging from −0.08 (West) to −0.58 μg m^−3^ (Southeast), while it overestimates it in the Northern Rockies (MB = 0.07 μg m^−3^) and Southwest (MB = 0.01 μg m^−3^). Correlation coefficients for SO_4_ are also generally strong (*r*^2^ > 0.80), with exceptions in the Southwest and West (*r*^2^ = 0.59 and 0.31, respectively). The EQUATES simulations reproduce the decreasing trend in observed concentrations with strong correlations (*r*^2^ > 0.79) (Fig. S7) but overpredict trends of SO_2_ and TNO_3_ concentrations and underpredicts trends of NH_4_ and SO_4_ concentrations.

### 3.2 Trend analysis

#### 3.2.1 Total deposition trends

**Figure 5.**
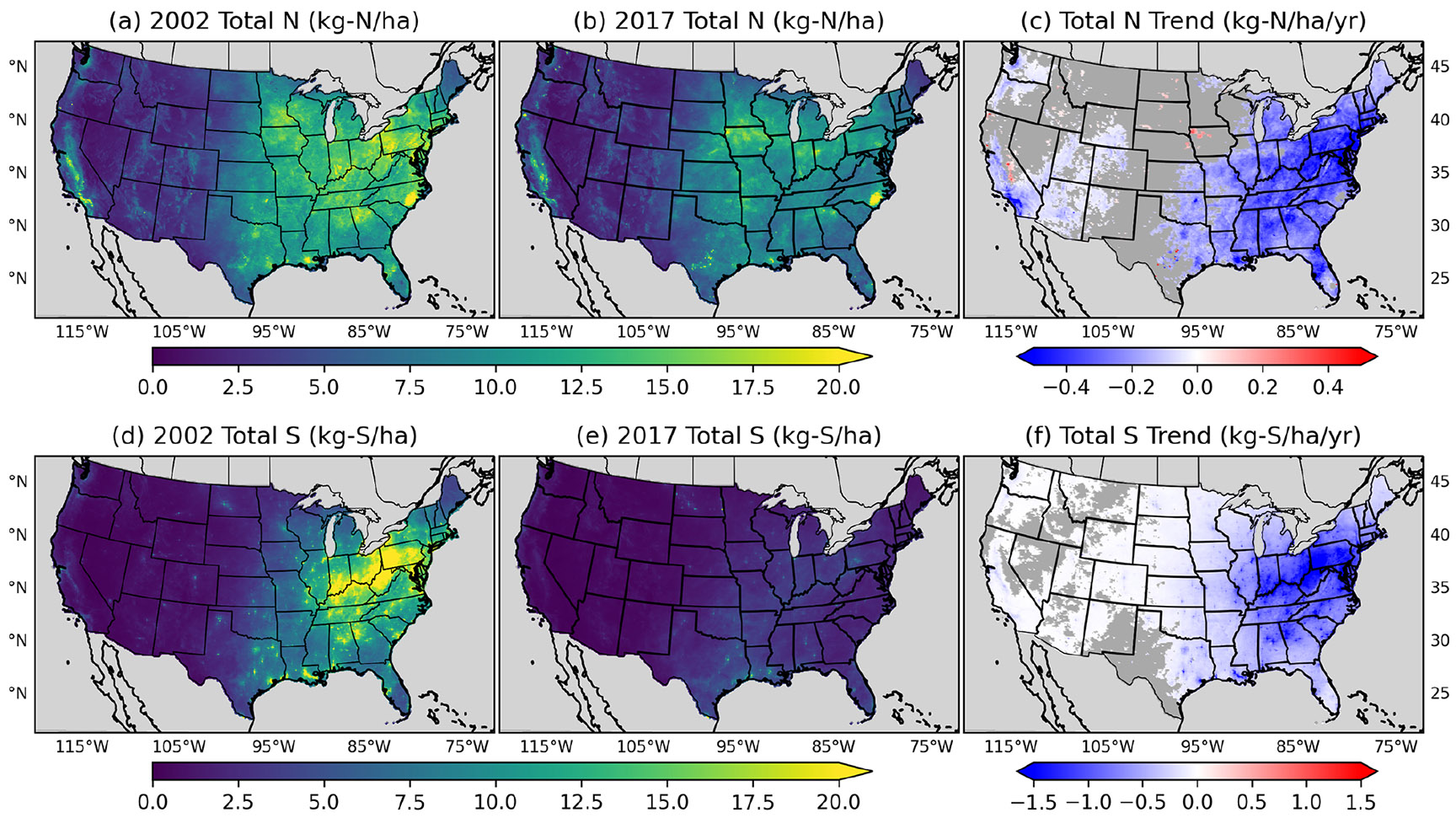
Spatial distribution of total N (**a, b, c**) and S (**d, e, f**) deposition in 2002 (**a** and **d**, kg ha^−1^) and 2017 (**b** and **e**, kg ha^−1^) and the 2002–2017 annual trend (**c** and **f**, kg ha^−1^ yr^−1^) with significance at the 95 % confidence level. Grey areas in panels (**c**) and (**f**) indicate where the trend is unavailable due to a lack of PRISM data or not significant (i.e., *p* value of the Wald test is greater than 0.05).

Figure 5 shows the spatial distribution and overall trend of modeled total deposition of N (i.e., the sum of oxidized and reduced N) and S between 2002 and 2017. For the trend analysis shown here and throughout, we calculate a linear least-squares regression with significance examined at the 95 % confidence level using a Wald test, with any insignificant trends set to zero. The eastern US has higher total N deposition amounts than the western US in both 2002 and 2017, particularly in parts of the Ohio Valley and Upper Midwest, regions associated with large N emissions from agriculture (Dammers et al., 2019) and energy consumption (Kim et al., 2006). The major N deposition region shifts from the eastern to the central US between 2002 and 2017, with highest deposition amounts found in parts of Iowa, North Carolina, and Indiana, a pattern consistent with N inventories compiled by Sabo et al. (2019) indicating an increase in agricultural fertilizer and livestock waste in the Midwest between 2002 and 2012. Increases in agricultural fertilizer have previously been linked to cropland expansion in the Great Plains (Lark et al., 2015; Wright et al., 2017) and increasing demand for domestically sourced biofuels (Donner and Kucharik, 2008). Urban regions in the central and eastern US indicate a substantial amount of N deposition compared to nearby rural areas (Fig. S8), consistent with previous findings that bulk N deposition in urban areas is twice as much as rural and remote sites (Decina et al., 2019).

Similar to total N deposition, total S deposition has a noticeable spatial gradient in the east compared to the west, particularly in the beginning of the time series, following larger changes in emissions in these regions (Aas et al., 2019; Holland et al., 1999). In 2002, total S deposition across the Southeast, Ohio Valley, and Northeast is broadly greater than 10 kg S ha^−1^, whereas S deposition for most of the western and central US climate regions is less than 5 kg S ha^−1^. By 2017, total S deposition continues to be highest in the eastern US, although total S deposition has decreased to less than 7.5 kg ha^−1^ on average. As the primary ion in acid deposition, S has been shown to reduce the biodiversity of natural ecosystems (Clark et al., 2019) but is also an essential macro-nutrient required for crop yield and health (Hinckley et al., 2020). Model projections spanning a range of future climate emission scenarios at the end of the century project global S deposition to agricultural soils to decrease by 70 %–90 % compared to 2005–2009 (Feinberg et al., 2021). Consequences of declining S deposition under growing food demand include risk of S deficiencies unless mitigating fertilizer strategies are developed and implemented. The spatial changes in decreasing total N and S deposition track changes in SO_2_ and NO_*x*_ emissions since 1970 (Nopmongcol et al., 2019), demonstrating the success of air quality mitigation policies implemented to achieve the National Ambient Air Quality Standards (NAAQS) under the CAAA (United States Congress, 1990) in also reducing nutrient and acid deposition. Concentrations of SO_2_, a primarily directly emitted compound, indicate greater decreasing trends than the secondary formed SO_4_. The non-linear relationship between SO_2_ and SO_4_ trends is shown in both the measurements and model output, as well as noted in other studies using several global CTMs (Aas et al., 2019) and monitoring data (Sickles and Shadwick, 2015), which attributed the increasing oxidative capacity of the atmosphere more efficiently converting SO_2_ to SO_4_ (Sickles and Shadwick, 2015). As SO_2_ emissions decline from coal-burning power plants due to controls implemented to meet the NAAQS (United States Congress, 1990), additional oxidants are available to oxidize SO_2_. The decline in SO_2_ emissions accompanied by near-constant NH_3_ emissions results in less acidic cloud droplets (Redington et al., 2009; Pye et al., 2020), which increases the oxidation rate of SO_2_ via the ozone pathway (Paulot et al., 2017). Lastly, the trends in wet deposition of SO_4_ (Fig. S6) are larger than the trends in concentration (Fig. S7), as SO_4_ is more efficiently scavenged by precipitation.

**Figure 6.**
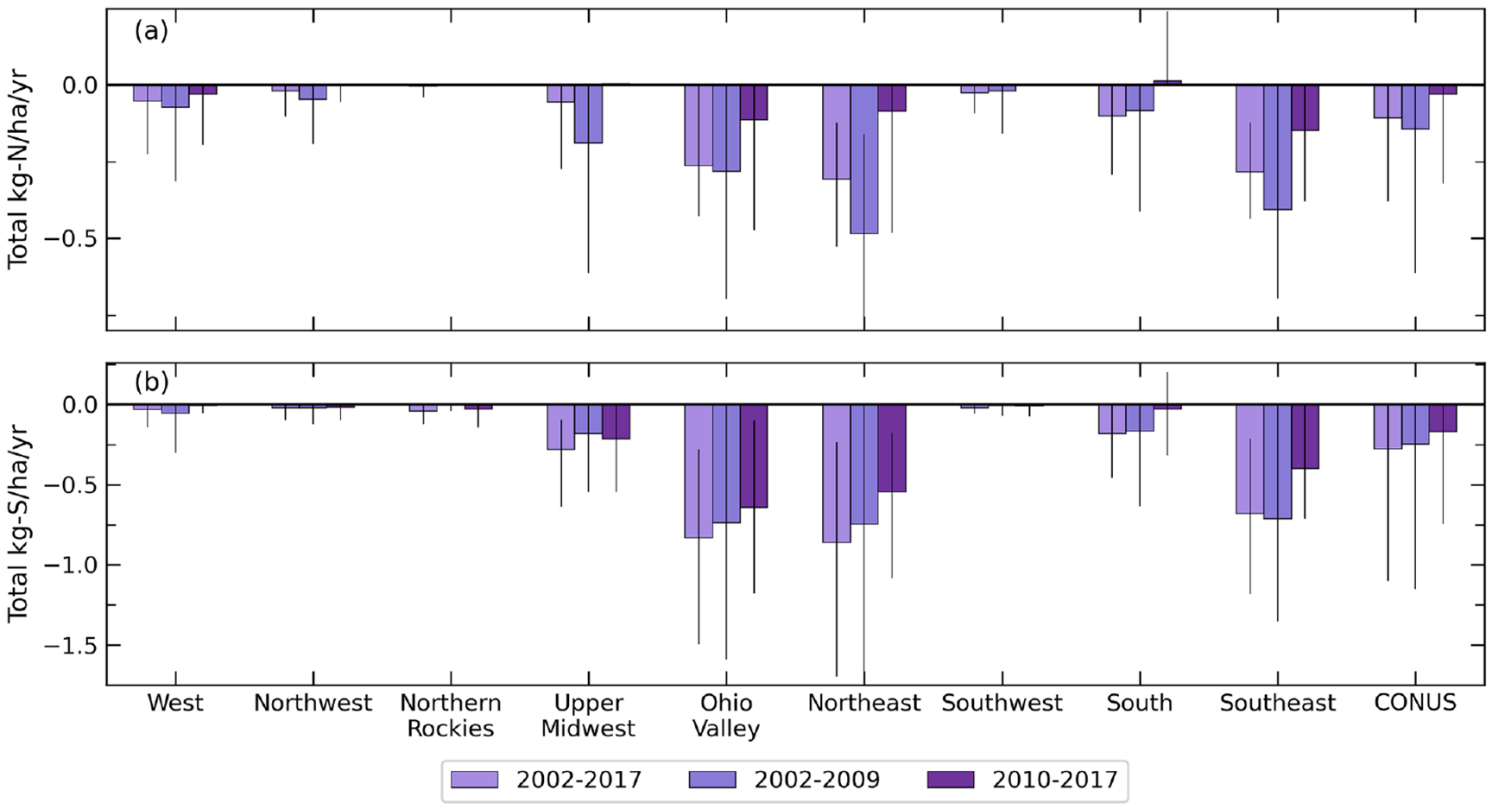
Comparison of mean total N (**a**) and total S (**b**) deposition trends in the nine CONUS climate regions from 2002–2017, 2002–2009, and 2010–2017. Lines on the bars extend to the 5th and 95th percentiles of the trends. Trends include all grid cell locations (insignificant trends are set to 0).

Figure 6 summarizes average total N and S trends across the climate regions and CONUS based on three different time bins: 2002–2017, 2002–2009, and 2010–2017. While there are significant regional deposition composition changes from 2002–2017, smaller changes in the overall deposition trends are observed for the CONUS. From 2002 to 2017, the largest average trend in decreasing total N deposition (−0.27 to −0.31 kg N ha^−1^ yr^−1^) occurs in the Ohio Valley, Northeast, and Southeast. Similarly, the Ohio Valley, Northeast, and Southeast experience the fastest rate of total S deposition decline from 2002 to 2017 (−0.68 to −0.86 kg S ha^−1^ yr^−1^), while the West, Northwest, Northern Rockies, and Southwest indicate near-zero (−0.03 kg Sha^−1^ yr^−1^) rates of decline. In many regions, especially the Northeast, Ohio Valley, and Southeast, a larger decreasing trend in total N and S deposition is found between 2002–2009 than 2010–2017. The high amount of total N and S deposition found in these regions combined with the slower rate of decline in recent years suggests additional emission controls may be necessary to continue deposition declines. On the other hand, the South is the only region with a very small increasing trend of ~ 0.01 kg N ha^−1^ yr^−1^ of total N deposition with large variability from 2010–2017 but also indicates a larger declining trend of −0.08 kg N ha^−1^ yr^−1^ from 2002–2009. Similarly, a large decreasing trend in total N deposition is found in the Midwest from 2002–2009, while the trend is near zero from 2010–2017. Fertilizer use in the Midwest has only grown modestly (~ 1.3 % yr^−1^), so increases in total N deposition have been largely attributed to increasing reduced N deposition. Growing-reduced N deposition is a result of NH_3_ emissions increasing exponentially with temperature (except below freezing and where emissions are near 0) (Riddick et al., 2016) and by increasing the partitioning of NH_3_ remaining in the gas phase due to NO_*x*_ and SO_2_ emission reductions (Warner et al., 2017) and increasing importance of on-road mobile emissions of NH3 (Fenn et al., 2018). As the world’s demand for food grows and NH_3_ emissions from agriculture increase, models project increasing *N*_r_ deposition in many areas across the globe by 2050 (Paulot et al., 2013) and 2100 (Lamarque et al., 2011).

**Figure 7.**
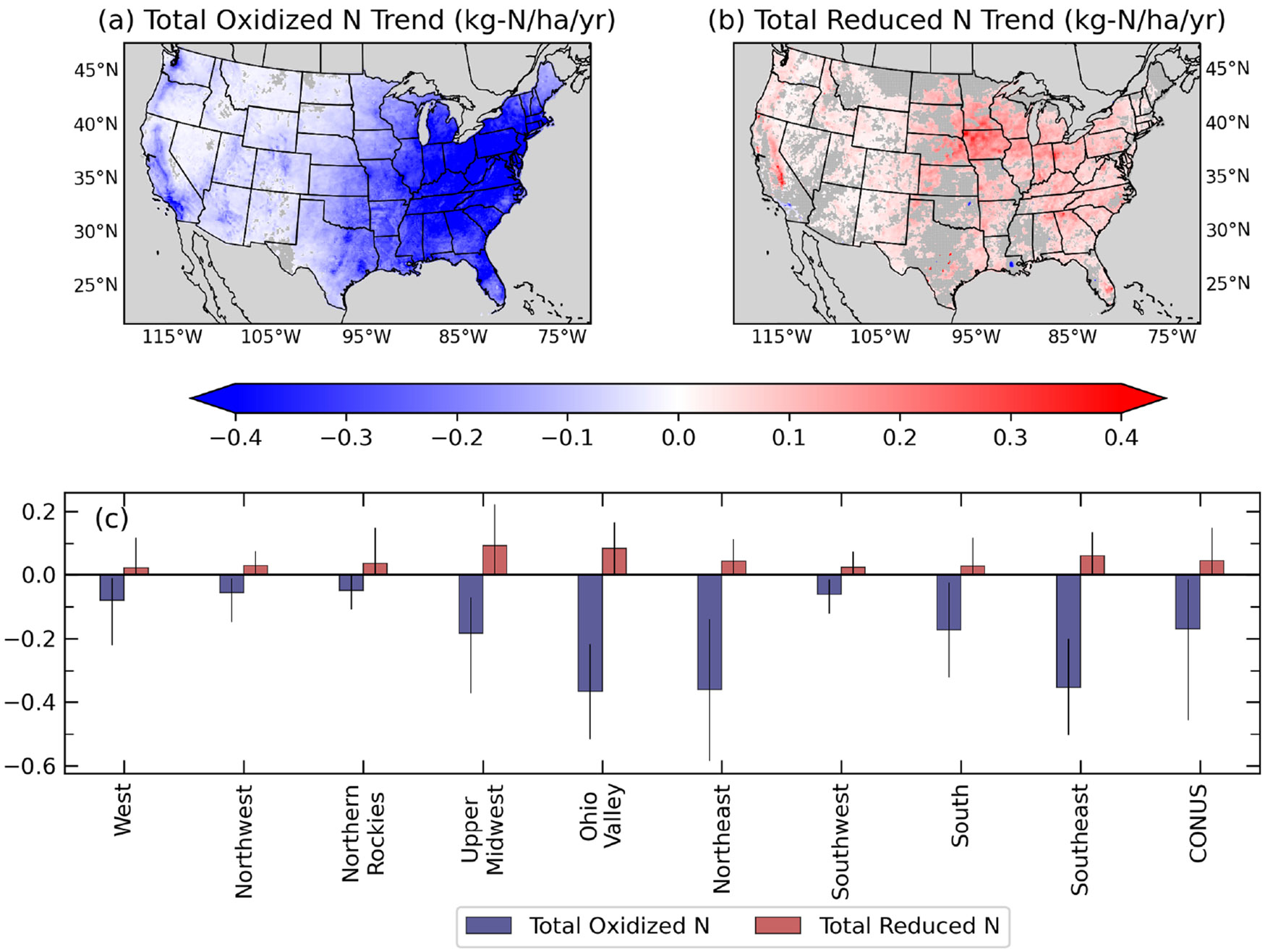
Maps of 2002–2017 trends of total oxidized (**a**) and reduced N deposition (**b**, kg N ha^−1^ yr^−1^) and comparison of average trends across nine CONUS climate regions (**c**). Lines in panel (**c**) show the 5th and 95th percentiles of the trends for each region. Grey colors on the map indicate where the trend is unavailable or insignificant.

To elucidate the chemical drivers responsible for the varying changes in total N deposition across the US, we examine trends in total oxidized and reduced N in Fig. 7. Overall, most regions show larger decreasing trends in oxidized N than increasing trends in reduced N. Regions with insignificant total trends (see grey areas in Fig. 5) indicate similar magnitudes of oxidized and reduced N trends. The largest decreasing trends in total oxidized N are found in the eastern US and along the Pacific coast. The large declines in total oxidized N in the Upper Midwest, Ohio Valley, Northeast, and Southeast showcase both the coordinated application of emission controls in major contributing regions and increasingly lower amounts of NO_*x*_ transported from source to receptor areas (Lloret and Valiela, 2016). Unlike trends of total N deposition (Fig. 5), statistically significant declining trends for total oxidized N are found throughout most of the CONUS, including the western US. No east–west spatial gradient is observed for trends in total reduced N deposition. Instead, reduced N deposition increases throughout most of the US, particularly in the Upper Midwest and Ohio Valley at an average rate of 0.09 kg N ha^−1^ yr^−1^, but is masked by the larger decreasing oxidized N trends. Declines in total reduced N deposition are located in Los Angeles, California; Tulsa, Oklahoma; and Baton Rouge, Louisiana, suggesting decreases in NH3 emissions near these cities. High NH_3_ deposition in Baton Rouge (> 10 kg ha^−1^) has been attributed to ammonia plant operations in the area (Guo et al., 2018), while dairy farms (Nowak et al., 2012) and vehicles (Cao et al., 2021) are major sources of NH_3_ in Los Angeles.

#### 3.2.2 Wet- and dry-deposition trends

**Figure 8.**
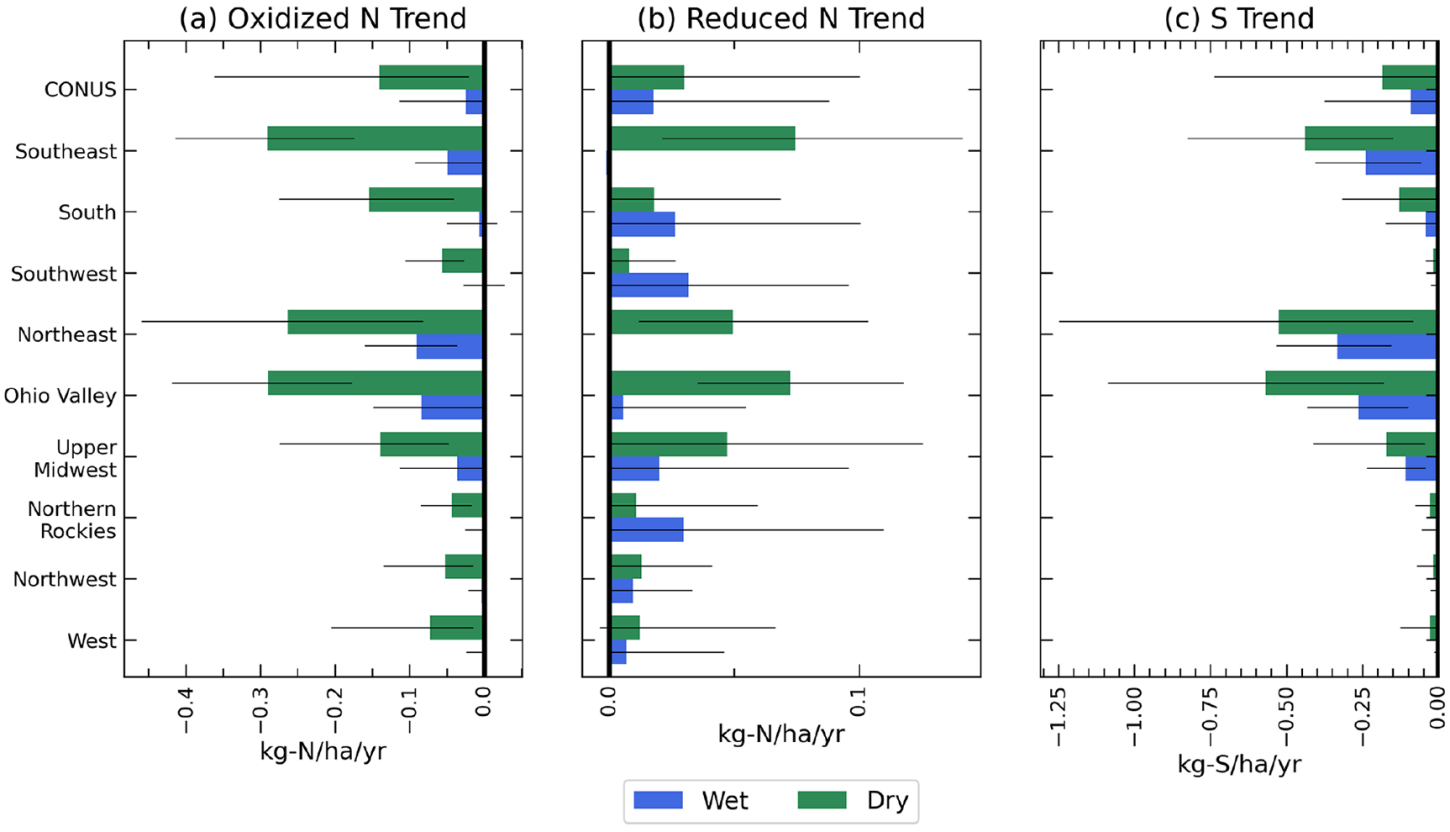
Annual average trend (2002–2017, kg ha^−1^ yr^−1^) of wet (blue) and dry (green) oxidized nitrogen (**a**), reduced nitrogen (**b**), and sulfur (**c**) deposition throughout the CONUS and nine climate regions. Whiskers extend to the 5th and 95th percentiles of the trends.

Figure 8 shows trends of the wet and dry components of oxidized and reduced N and S deposition. Average dry deposition of oxidized N is found to decline faster than average wet deposition of oxidized N for all climate regions, as oxidized N is more efficiently removed by dry deposition due to high deposition velocities (Zhang et al., 2012). Larger amounts of dry vs. wet deposition across the eastern US in 2002 (Fig. S9a and d) and more comparable amounts in 2017 (Fig. S9b and e) suggest a shift over time in the contributions of oxidized wet and dry deposition, although the portioning is highly dependent upon annual rainfall and changes to climate. The Southeast, Northeast, and Ohio Valley have the steepest simulated average dry and wet oxidized N deposition trends (approximately −0.3 and −0.1 kg N ha^−1^ yr^−1^, respectively), consistent with the observed trends (Fig. S10). The NTN sites with the highest mean NO_3_ deposition, particularly those in the Northeast, experience the largest decreasing trends between 2002–2009 (−2.2kg N ha^−1^ yr^−1^) compared to 2010–2017 (−1.0kg N ha^−1^ yr^−1^), consistent with the trends shown in Fig. 6.

Trends of reduced N are increasing and overall smaller in magnitude compared to the oxidized N trends (Fig. 7b). Regions of elevated wet and dry reduced N deposition have expanded and increased in magnitude across the CONUS (Fig. S11) compared to oxidized N, also observed in the NTN NH_4_ measurements. The increasing trends of NH_4_ wet deposition accompany statistically significant increasing trends in annual precipitation predominantly over the Upper Midwest, Southwest, and Northern Rockies (Fig. S12). Similar increasing trends of NH_4_ wet deposition between 1989 and 2016 observed in the Midwest and Mid-Atlantic have also been linked to rising annual precipitation amounts (Feng et al., 2021). Similarly, NTN sites in the Upper Midwest with the highest mean NH_4_ wet deposition experience larger increasing trends from 2002 to 2017 (Fig. S10). Similar statistically significant increases in NH_4_ wet-precipitation concentrations have been reported, although increases are largest for the period prior to our model simulations (1985–1999) (Mchale et al., 2021). The decreasing reduced N deposition in the three urban centers discussed in Sect. 3.2.1 is present in the dry-deposition trends but not in wet deposition. The Southeast and Northeast have a near-zero trend in wet reduced N deposition but a larger increasing trend in dry reduced N deposition. Parts of the Southeast, including southwestern Florida, experience a decrease in precipitation across the entire period, which may partially explain the decreasing trend of wet reduced N deposition.

Decreasing trends in wet and dry S deposition are found across the CONUS, although the amount varies by climate region (Figs. 8c, S13). Similar to the oxidized N trends, the Northeast, Southeast, and Ohio Valley experience the largest decreasing trend of approximately −0.5 kg S ha^−1^ yr^−1^ for dry deposition and −0.3 kg S ha^−1^ yr^−1^ for wet deposition, consistent with the NTN SO_4_ observations (Fig. S10). Sites with the largest mean SO_4_ wet deposition in the Ohio Valley and Northeast also experience the largest decreasing trends in wet SO_4_ deposition (−1.9kg S ha^−1^ yr^−1^, Fig. S10), consistent with monthly mean precipitation-weighted concentrations from NTN sites from 1985 to 2017 (Mchale et al., 2021). Large decreasing trends in dry S deposition are clearly seen over large urban centers in the western US, while the eastern US experiences a significant declining trend everywhere in response to decreases in regional SO_2_ emissions (Likens et al., 2001).

### 3.3 Total nitrogen and sulfur deposition budgets

**Figure 9.**
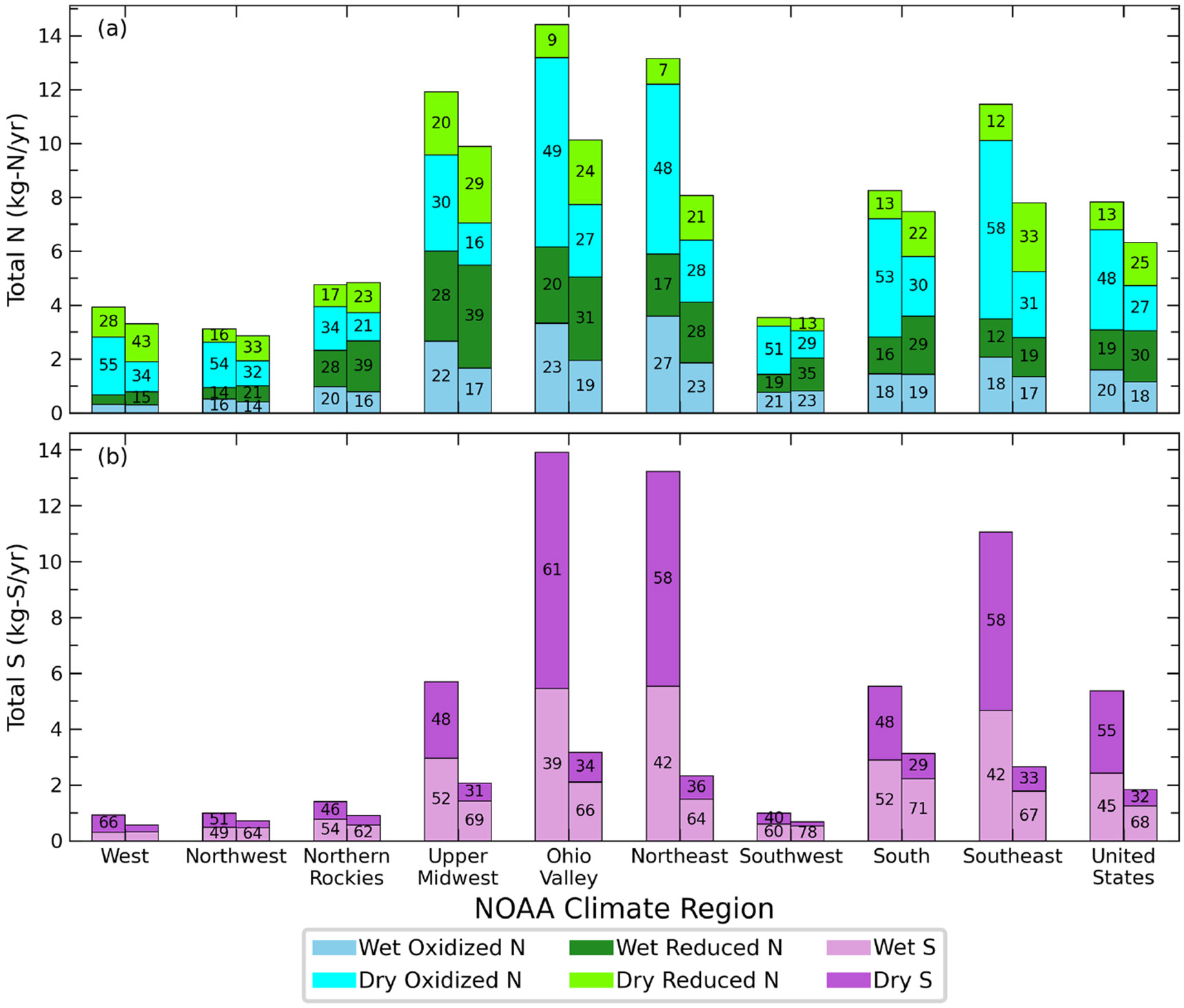
Average total (wet + dry) N (**a**) and S (**b**) deposition in 2002 (left bars) and 2017 (right bars) throughout the nine climate regions and CONUS. Colors denote the wet and dry component of each species. The numbers in each bar denote the percentage contribution to the annual total.

The average N and S deposition budgets across all CONUS climate regions generally decrease from 2002 to 2017 (Fig. 9). The average total N budget for the CONUS decreases from 7.8 to 6.3 kg N ha^−1^ yr^−1^ between 2002 and 2017. The Upper Midwest, Ohio Valley, Northeast, and Southeast have the largest average total N, decreasing from 12–14 in 2002 to 8–10 kg N ha^−1^ yr^−1^ in 2017. In contrast, the Northwest, West, and Southwest have the smallest average total N, which decreases only slightly from 3.1–3.9 to 2.8–3.5 kg N ha^−1^ yr^−1^ over the same time period. The decrease in total N deposition is largely due to reductions in oxidized N, particularly from dry deposition, and declines in NO_x_ emissions (Nopmongcol et al., 2019). The average wet oxidized N deposition for the CONUS decreases from 1.6 in 2002 to 1.1 kg N ha^−1^ yr^−1^ in 2017, while the dry component decreases from 3.7 to 1.7 kg N ha^−1^ yr^−1^. Dry deposition constitutes most of the total N budget (slightly decreasing from 50 %–82 % in 2002 to 42 %–76 % in 2017 across the climate regions), with the largest average contributions found in the western US. Spatial differences in the dry N deposition flux, inferred using Ozone Monitoring Instrument (OMI) satellite observations of NO2 and ground measurements from 2005 to 2014, indicate an increasing flux across the western US and decreasing flux over the eastern US due to reductions in NO_*x*_ and NH_3_ emissions (Jia et al., 2016). Unlike oxidized N, the wet reduced N deposition is larger on average than the dry deposition for all regions except the West and Northwest, regions that typically experience less rainfall than the other regions. Similar to Du et al. (2014), we find an increasing contribution of wet reduced N deposition to the total across the CONUS from 19 % to 30 % across the time period (Fig. S14).

**Figure 10.**
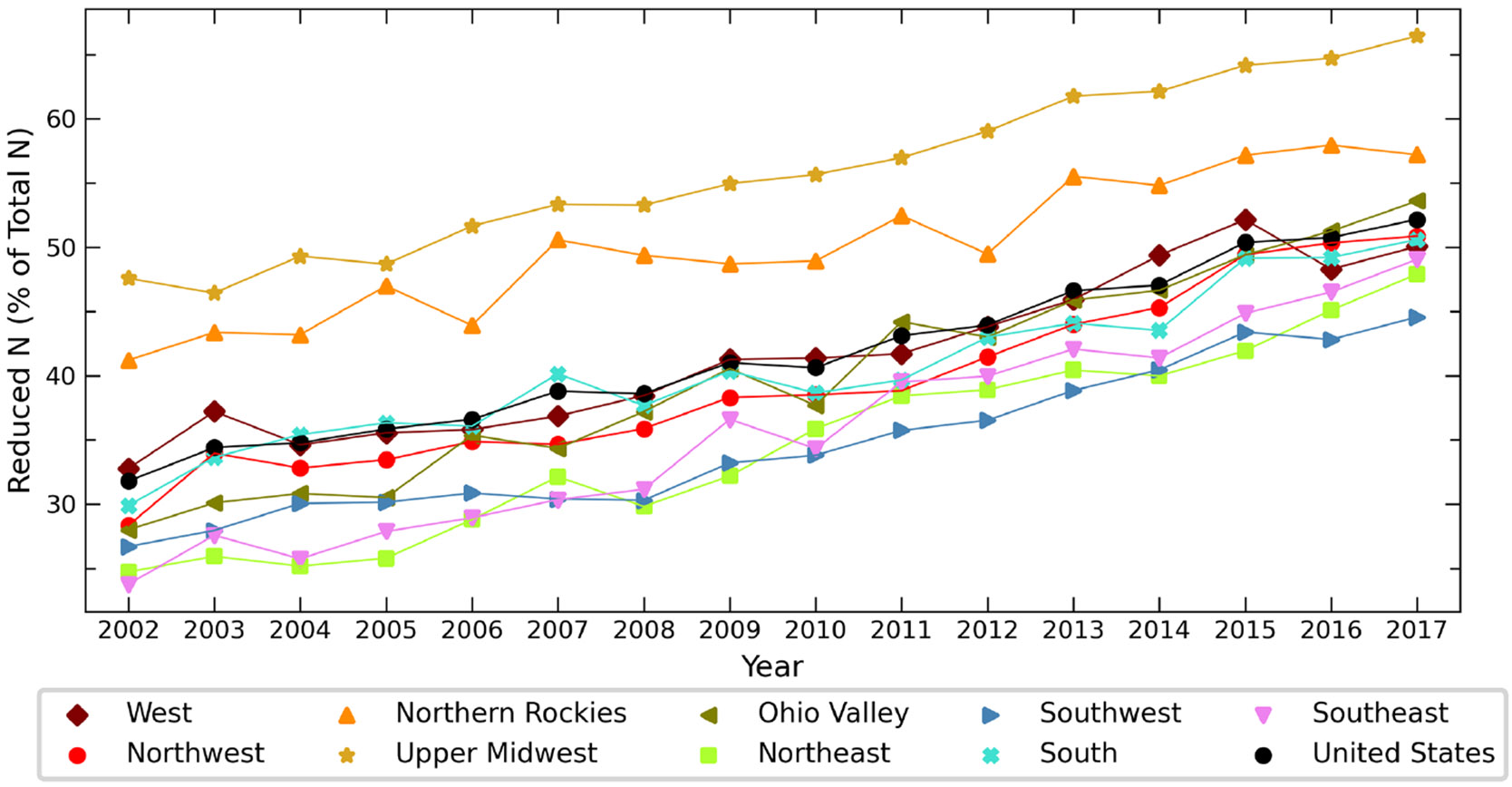
Changes in the percent of total N deposited as reduced nitrogen from 2002 to 2017 throughout the NOAA climate regions and CONUS.

The relative proportion of reduced N to the total budget has shifted over the time period of 2002 to 2017 (Fig. 10). On average across the CONUS, oxidized N deposition dominates the total budget until 2016, when reduced N comprises > 50 % of the total, consistent with previous studies (Du et al., 2014; Kanakidou et al., 2016; Ackerman et al., 2019; Li et al., 2016; Nopmongcol et al., 2019) and attributable to the absence of NH3 emission controls from agricultural sources combined with the success of NO_*x*_ regulations. The reductions in NO_*x*_ and SO_*x*_ emissions due to policy controls result in less NH_*x*_ partitioning to the aerosol phase and more to the gas phase, favoring dry deposition closer to source regions. Despite spatial variations in agricultural NH_3_ sources and a lack of an emission trend during this study period over some locations, all climate regions indicate an increasing amount of reduced N to the total budget, agreeing with results from Nopmongcol et al. (2019). In order to protect critical ecosystems within US national parks from future N deposition, Ellis et al. (2013) project at least a 50 % reduction in anthropogenic NH_3_ emissions is required relative to RCP-projected (Representative Concentration Pathway) emission scenario levels in 2050. The fraction of reduced N to the total is largest in regions with larger NH_3_ emissions (Dammers et al., 2019; Behera et al., 2013), such as the Upper Midwest and Northern Rockies, throughout the time period studied here. These regions exceed 50 % of reduced N as a fraction of the total budget as early as 2006 for the Upper Midwest and 2011 for the Northern Rockies. Approximately 80 % of the total global NH_3_ emissions in 2005 were attributable to agriculture, including animal feedlot operations, and are anticipated to increase in the US and globally (Behera et al., 2013). Present-day (2015) global simulations indicate substantial regional variation in the *N*_r_ budget, reflecting the impact of regional activities and meteorology (Ge et al., 2022). The increasing importance of reduced N is likely to impact future N deposition budgets and the competitive nature among plants with varying affinities for the different forms of nitrogen (Choudhary et al., 2016; Kahmen et al., 2006).

The total S budget also decreases across the CONUS on average, falling from 5.3 in 2002 to 1.8 kg S ha^−1^ yr^−1^ in 2017. Decreasing SO_2_ emissions have been previously shown to be responsible for decreasing S deposition (Nopmongcol et al., 2019). Similar to the total N budget, the largest average amounts of total S are located in the Ohio Valley, Northeast, and Southeast, decreasing from 11–14 in 2002 to 8–10 kg S ha^−1^ yr^−1^ in 2017. In the West, Ohio Valley, Northeast, and Southeast, dry deposition dominates the budget for 2002, while transitioning wet deposition dominated by 2017. Wet deposition comprises a greater portion of the total S budget in 2017 across all climate regions. The primary driver behind the shift in the total S budget may be emission reductions in SO_2_ implemented to meet the NAAQS (leading to decreasing ambient concentrations of SO_2_ and SO_4_) that more efficiently decrease the dry component than the wet (Sickles and Shadwick, 2015).

## 4 Conclusions

In this study, we examine CMAQ model simulations from the EPA’s Air QUAlity TimE Series (EQUATES) project to investigate spatial and temporal trends of nitrogen (N) and sulfur (S) deposition over a period with substantial emission reductions implemented to meet requirements set by the Clean Air Act Amendments (CAAA). We assess changes in modeled dry deposition and precipitation- and bias-adjusted wet deposition across nine climatologically consistent regions within the US. EQUATES CMAQ simulations included important science updates to previous modeling studies, such as a revised model for bidirectional air–surface exchange of NH_3_ and a mechanistic representation of organic N (Pye et al., 2015), necessary to improve modeling of *N*_r_ budgets. The measurement–model fusion technique for modeled wet-deposition estimates reduces the model bias and improves correlations compared to the unadjusted model values and can thus improve the accuracy of critical-load assessments based on model data. Comparisons to wet-deposition observations from the NADP network indicate the model generally underestimates wet deposition of NH_4_, NO_3_, and SO_4_ across the CONUS. Agreement is poor in regions with complex terrain like the Northern Rockies and West, potentially due to errors constraining emissions and/or reproducing precipitation at 12 km grid spacing, as sub-grid variability in precipitation estimates can differ from sub-grid variability in concentrations across the country. Similarly, the model underestimates ambient concentrations of NH_4_ and SO_4_ compared to CASTNET measurements across the CONUS, while SO_2_ and TNO_3_ concentrations are overestimated. Additional measurements in regions with high bias, such as the West, Northwest, and Southwest, as well as model improvements to precipitation estimates, particularly in areas of complex terrain, could greatly improve agreement with the NADP network observations.

The modeled total N and S deposition amounts and trends are larger across the eastern than the western US, particularly in the Northeast, Southeast, Ohio Valley, and Upper Midwest climate regions. While the average CONUS trend indicates a decrease of −0.11 kg N ha^−1^ yr^−1^ in total N deposition from 2002–2017, significant increasing trends are found in localized parts of the Upper Midwest, Northern Rockies, and West. Such increases in total N deposition are dominated by increases in reduced N that are detected across all climate regions but are masked by the larger declining trends of oxidized N deposition as a result of policies targeting NO_*x*_ emissions enacted under the CAAA. The increasing trend in reduced N from 2002 to 2017 is a result of growing agricultural emissions combined with NO_*x*_/SO_*x*_ emission reductions and higher precipitation amounts over time concentrated in the Upper Midwest, Southwest, and Northern Rockies. Despite the overall average declining N trend from 2002 to 2017, the simulations indicate a larger average decreasing trend of total N and S at the beginning of the time series (2002 to 2009) than the end of the period (2010 to 2017). Total N trends in the South reveal a slight increasing trend (0.01 kg N ha^−1^ yr^−1^) from 2010 to 2017, contrary to the larger declining trends found between 2002–2009. The modeled slowing and slightly increasing trend for total N across the climate regions in the EQUATES simulations hints at future changes to the N budget that may impact plant biodiversity and productivity (Kahmen et al., 2006).

The average total N deposition budget over the CONUS has decreased from 7.8 in 2002 to 6.3 kg N ha^−1^ yr^−1^ in 2017, largely due to decreases in oxidized N deposition as a result of NO_*x*_ emission controls implemented under the CAAA. The major contributor to the N budget is dry deposition, although the contribution across the climate regions has declined from 50 %–82 % in 2002 to 42 %–76 % in 2017, in part due to changes in precipitation amounts. The regions with the largest average total N deposition, including the Ohio Valley, Northeast, Upper Midwest, and South, also experience the largest reduction in total N deposition. Similar to the total N budget, the average total S budget over the CONUS has declined from 5.3 in 2002 to 1.8 kg S ha^−1^ yr^−1^ in 2017. The largest amount of total S occurs in the eastern US, where total deposition is more impacted by dry deposition. By the end of the time series, wet deposition dominates the S budget for all climate regions, comprising 68 % of the total budget in the CONUS on average.

Our analysis, in addition to the analyses of many others (Zhang et al., 2018; Li et al., 2016; Du et al., 2014), highlights the increasing contribution of reduced compounds to the N budget in all climate regions, particularly the Northern Rockies and Upper Midwest. Both the model and observations indicate statistically significant increasing trends in NH_4_ deposition across the CONUS due to changes in precursor emissions. Reductions in NO_*x*_ and SO_2_ emissions, and therefore their oxidation products, due to policy controls result in less NH_*x*_ partitioning to the aerosol phase and more to the gas phase, favoring dry deposition of NH_3_ closer to source regions. Additionally, growth of NH_3_ emissions from agricultural sources (Behera et al., 2013) allows for additional NH_4_ aerosol formation in the atmosphere given sufficient NO_*x*_ and SO_*x*_. This study further confirms the growth of reduced N in recent years across all climate regions and additionally suggests an attenuation of the declining oxidized N deposition trend. However, since N deposition over urban areas across the CONUS is likely already underestimated (Rao et al., 2013), with increasing urbanization only expected to further increase N deposition amounts (Joyce et al., 2020), addressing modeling uncertainty in emissions and chemistry at relevant spatial and temporal resolutions is imperative. For instance, a more robust characterization of dry deposition in the US, including low-cost dry deposition and ambient measurement methods, is needed, particularly in agricultural areas and regions with transitions from urban to rural environments, as well as for species not routinely measured, such as organic compounds. In this regard, additional ambient NH_3_ measurements are of critical importance for improving the modeling and analytical methods used in air quality (and related ecosystem) management for particulate matter and N deposition.

## Supplementary Material

Supplement1

## Figures and Tables

**Table 1.** EQUATES model performance metrics of annual (2002–2017) accumulated wet deposition of NH_4_, NO_3_, and SO_4_, including the Pearson correlation coefficient (*r*^2^), mean bias (MB, kg ha^−1^), and normalized mean bias (NMB, %).

Metric	Wet-deposition correction	NH_4_	NO_3_	SO_4_
*r* ^2^	No adjustment	0.61	0.77	0.78
	Precipitation adjustment	0.68	0.79	0.83
	Bias adjustment	0.81	0.88	0.92
Mean bias (MB, kg ha^−1^)	No adjustment	−0.49	0.66	−0.92
	Precipitation adjustment	−0.37	1.18	−0.43
	Bias adjustment	−0.07	−0.03	−0.10
Normalized mean bias (NMB, %)	No adjustment	−19.9	9.54	−12.2
	Precipitation adjustment	−15.1	17.1	−5.73
	Bias adjustment	−2.83	−0.48	−1.31

**Table 2.** Evaluation of bias-adjusted SO_4_, NO_3_, and NH_4_ wet deposition and precipitation compared to NTN measurements in the contiguous US and nine climate regions that meet the completeness criteria (obs/mod: observed/modeled).

	Climate region	United States	Northeast	Northwest	Ohio Valley	Northern Rockies	South	Southeast	Southwest	Upper Midwest	West
	Sites (*N*)	200 (3076)	35 (531)	12 (184)	27 (418)	19 (284)	21 (323)	29 (447)	26 (400)	21 (330)	10 (150)
SO_4_	Mean obs/mod (kg ha^−1^)	7.50/7.40	11.48/11.16	2.53/2.45	13.1/13.1	2.71/2.48	7.82/7.89	9.82/9.70	2.22/2.23	6.65/6.53	1.16/1.32
	MB (kg ha^−1^)	−0.10	−0.32	−0.08	−0.03	−0.23	0.07	−0.11	0.01	−0.11	0.16
	NMB (%)	−1.31	−2.78	−3.26	−0.25	−8.37	0.87	−1.16	0.46	−1.71	14.1
	*r* ^2^	0.92	0.89	0.81	0.82	0.61	0.87	0.84	0.47	0.86	0.56
NO_3_	Mean obs/mod (kg ha^−1^)	6.90/6.87	9.87/9.79	2.51/2.50	10.33/10.44	4.07/3.68	6.91/7.01	7.53/7.43	3.70/3.86	8.00/7.83	1.80/1.91
	MB (kg ha^−1^)	−0.03	−0.08	−0.01	0.11	−0.39	0.09	−0.10	0.16	−0.18	0.11
	NMB (%)	−0.48	−0.80	−0.39	1.02	−9.49	1.37	−1.29	4.27	−2.24	6.01
	*r* ^2^	0.88	0.82	0.82	0.67	0.54	0.86	0.69	0.52	0.75	0.6
NH_4_	Mean obs/mod (kg ha^−1^)	2.43/2.36	2.64/2.56	0.82/0.73	3.57/3.59	2.13/2.02	2.82/2.74	2.31/2.16	1.20/1.16	3.82/3.76	0.87/0.81
	MB (kg ha^−1^)	−0.07	−0.08	−0.09	0.03	−0.11	−0.08	−0.15	−0.04	−0.06	−0.07
	NMB (%)	−2.83	−3.06	−10.6	0.76	−5.17	−2.69	−6.32	−2.93	−1.45	−7.72
	*r* ^2^	0.81	0.68	0.52	0.66	0.89	0.64	0.59	0.45	0.77	0.34
Precipitation	Mean obs/mod (cm)	96.7/94.4	122.1/116.1	124.5/136.5	116.9/118.2	57.4/64.8	97.0/82.6	128.7/111.0	49.2/58.7	82.0/83.2	53.2/50.6
	MB (cm)	−2.32	−6.05	12	1.32	7.4	−14.4	−17.7	9.45	1.22	−2.64
	NMB (%)	−2.40	−4.95	9.62	1.13	12.9	−14.9	−13.7	19.2	1.49	−4.97
	*r* ^2^	0.72	0.27	0.87	0.36	0.42	0.76	0.28	0.65	0.5	0.81

**Table 3.** Evaluation of EQUATES modeled average concentrations (μg m^−3^) of SO_2_, SO_4_, TNO_3_, and NH_4_ compared to CASTNET measurements in the CONUS and eight climate regions (no CASTNET data are available in the Northwest climate region; obs/mod: observed/-modeled).

	Climate region	United States	Northeast	Ohio Valley	Northern Rockies	South	Southeast	Southwest	Upper Midwest	West
	Sites (*N*)	75 (1175)	15 (237)	18 (284)	5 (79)	6 (94)	12 (185)	8 (126)	5 (78)	6 (92)
SO_4_	Mean obs/mod (μg m^−3^)	2.15/1.82	2.40/2.06	3.16/2.66	0.55/0.62	2.37/1.86	2.73/2.16	0.61/0.61	1.77/1.64	0.78/0.70
	MB (μg m^−3^)	−0.33	−0.34	−0.50	0.07	−0.50	−0.58	0.01	−0.13	−0.08
	NMB (%)	−15.4	−14.3	−15.9	12.8	−21.2	−21.1	1.34	−7.20	−10.9
	*r* ^2^	0.96	0.97	0.93	0.82	0.92	0.93	0.59	0.97	0.31
SO_2_	Mean obs/mod (μg m^−3^)	2.23/2.81	3.38/4.40	3.81/4.69	0.47/0.44	1.05/1.47	2.10/2.68	0.36/0.34	1.68/2.50	0.40/0.21
	MB (μg m^−3^)	0.58	1.02	0.88	−0.03	0.41	0.58	−0.02	0.82	−0.19
	NMB (%)	26	30.3	23.2	−6.95	39.3	27.6	−5.61	49.1	−47.3
	*r* ^2^	0.9	0.91	0.85	0.37	0.78	0.86	0.76	0.96	0.09
NH_4_	Mean obs/mod (μg m^−3^)	0.78/0.64	0.87/0.72	1.23/1.01	0.22/0.20	0.74/0.63	0.81/0.67	0.22/0.18	0.83/0.75	0.31/0.19
	MB (μg m^−3^)	−0.14	−0.15	−0.22	−0.02	−0.11	−0.14	−0.04	−0.08	−0.11
	NMB (%)	−17.4	−17.8	−17.8	−8.78	−14.8	−17.4	−18.7	−10.1	−37.2
	*r* ^2^	0.94	0.91	0.92	0.83	0.85	0.92	0.39	0.95	0.38
TNO_3_	Mean obs/mod (μg m^−3^)	1.72/1.77	1.77/2.01	2.52/2.79	0.55/0.57	1.69/1.59	1.56/1.48	0.79/0.78	1.98/2.06	1.55/0.91
	MB (μg m^−3^)	0.05	0.24	0.26	0.02	−0.10	−0.07	−0.01	0.09	−0.64
	NMB (%)	2.8	13.6	10.5	4.23	−5.98	−4.76	−1.35	4.34	−41.6
	*r* ^2^	0.81	0.79	0.86	0.87	0.9	0.4	0.59	0.95	0.79

## Data Availability

Data are available for download at https://www.epa.gov/cmaq/equates and https://www.epa.gov/cmaq/data-download-step-2?token=It6R0_m8gdFff3WHM3wFaSvIgsXjaToYLMQa6fc5TsA (EPA, 2022).
