# Supplementary material for: Long-term regional trends of nitrogen and sulfur deposition in the United States from 2002 to 2017
Source: Atmos Chem Phys. Author manuscript; Available in PMC 2025 Feb 26. (PMC11864274; doi:10.5194/acp-22-12749-2022)
Supplement: Supplement1 [file NIHMS2041242-supplement-Supplement1.pdf]

Supplement of Atmos. Chem. Phys., 22, 12749–12767, 2022
https://doi.org/10.5194/acp-22-12749-2022-supplement
© Author(s) 2022. CC BY 4.0 License.

Atmospheric Chemistry and Physics
Open Access

Supplement of

Long-term regional trends of nitrogen and sulfur deposition in the United States from 2002 to 2017

Sarah E. Benish et al.

Correspondence to: Sarah E. Benish (benish.sarah@epa.gov) and Jesse O. Bash (bash.jesse@epa.gov)

The copyright of individual parts of the supplement might differ from the article licence.

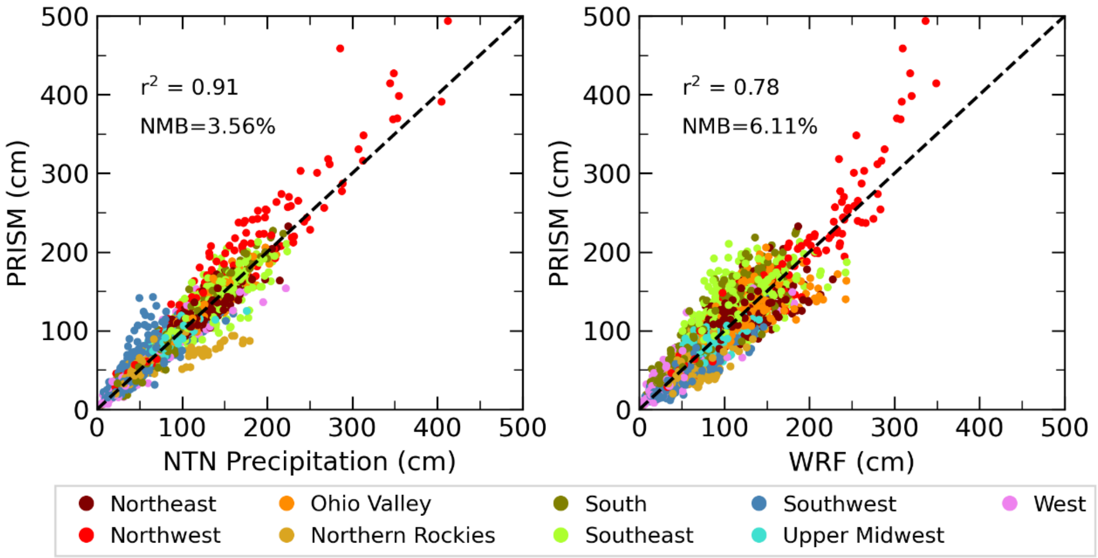

Figure S1. Left: Scatter plot of annual accumulated precipitation (cm) from PRISM and observed at selected NTN sites, colored by the NOAA climate region. Right: Scatter plot of annual accumulated precipitation (cm) modeled in CMAQ (WRF) and estimated from PRISM, colored by the NOAA climate region. The positive normalized mean bias indicates the PRISM precipitation amounts are larger than the NTN or WRF precipitation amounts.

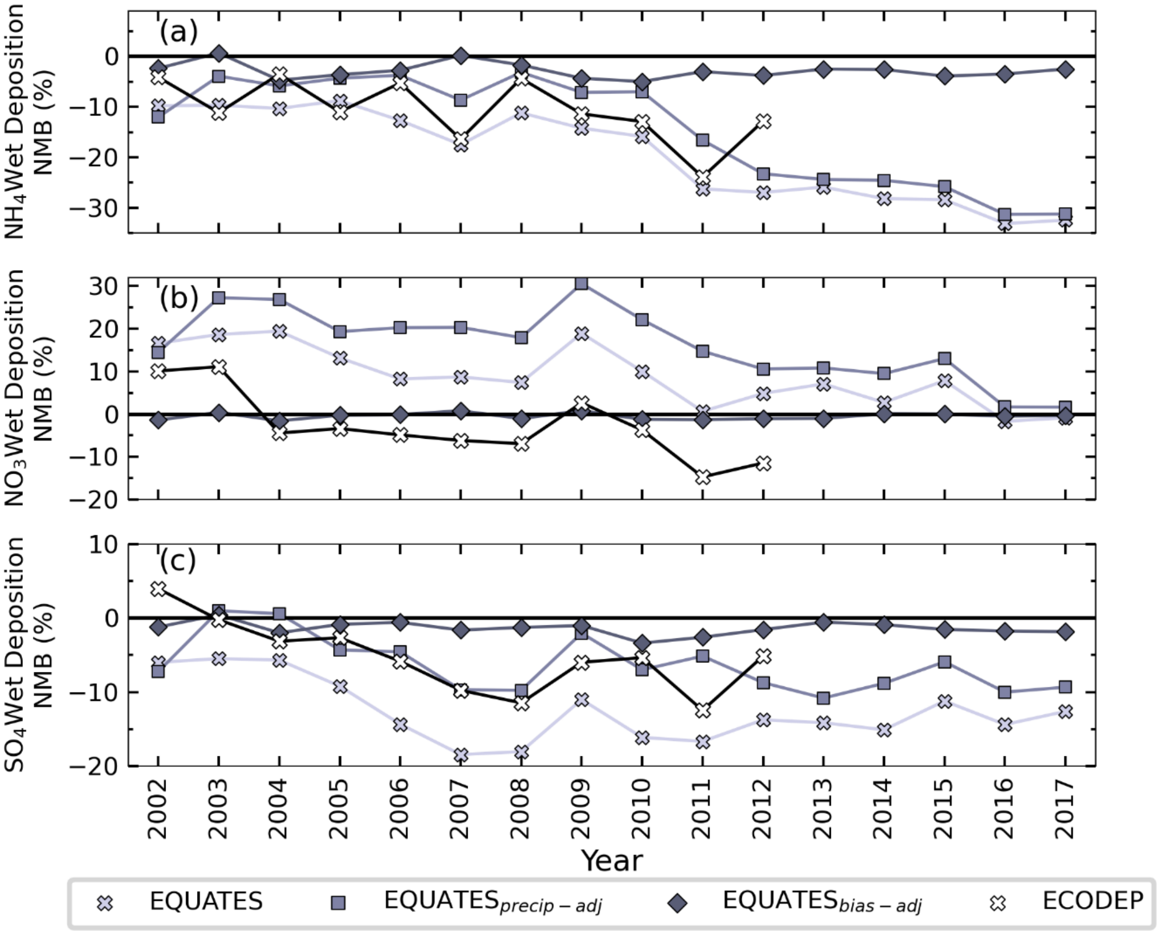

Figure S2. Annual normalized mean bias (NMB, %) for unadjusted, precipitation-adjusted, and bias-adjusted modeled NH_4 (panel a), NO_3 (panel b), and SO_4 (panel c) wet deposition compared to NTN measurements.

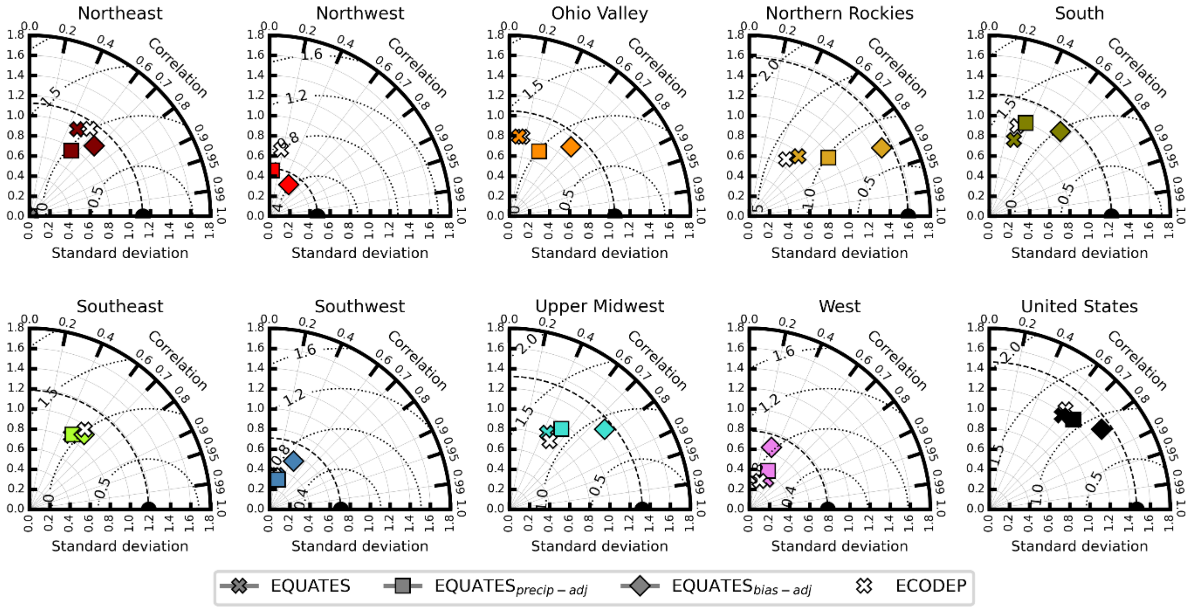

Figure S3. Taylor plot comparing 2002-2017 annual accumulated measured NH_4 wet deposition (kg/ha) with EQUATES and ECODEP (2002-2012) model output across climate regions within the CONUS. The symbols show how the unadjusted (x's), precipitation-adjusted (squares) and bias-adjusted (diamonds) modeled wet deposition compare to the NTN measurements (black circles).

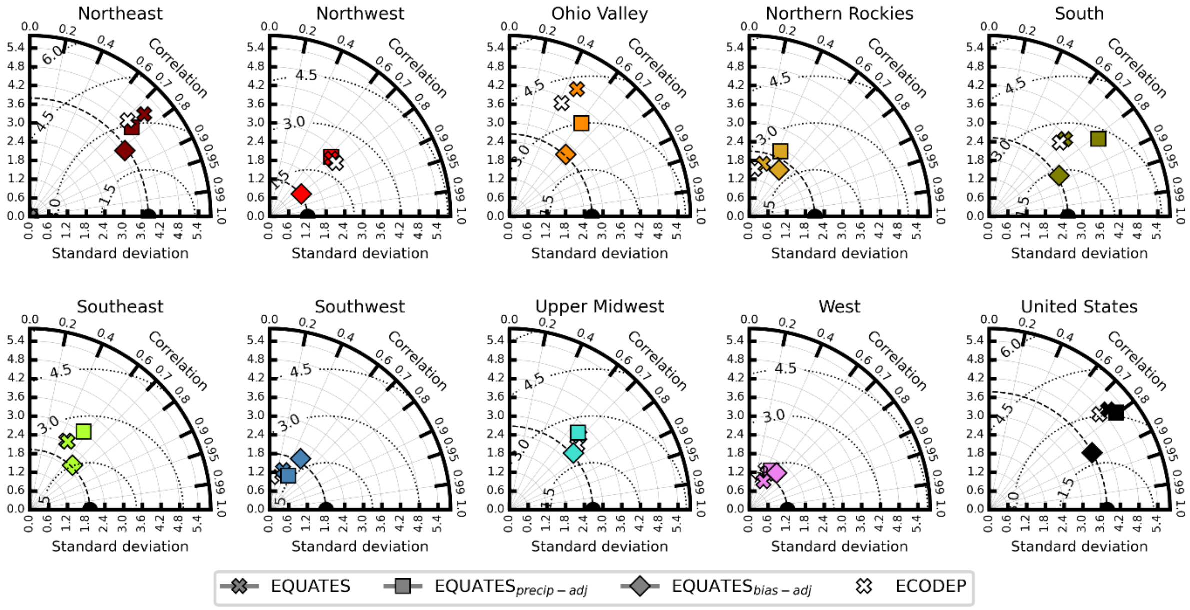

Figure S4. Taylor plot comparing 2002-2017 annual accumulated measured NO_3 wet deposition (kg/ha) with EQUATES and ECODEP (2002-2012) model output across climate regions within the CONUS. The symbols show how the unadjusted (x's), precipitation-adjusted (squares) and bias-adjusted (diamonds) modeled wet deposition compare to the NTN measurements (black circles).

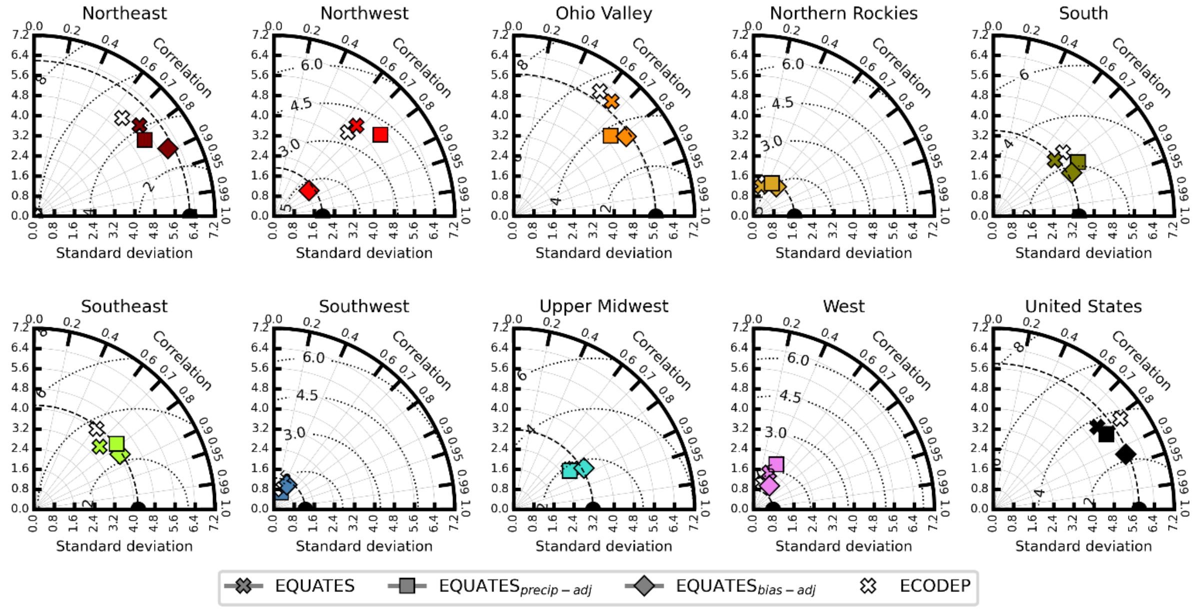

Figure S5. Taylor plot comparing 2002-2017 annual accumulated measured SO_4 wet deposition (kg/ha) with EQUATES and ECODEP (2002-2012) model output across climate regions within the CONUS. The symbols show how the unadjusted (x's), precipitation-adjusted (squares) and bias-adjusted (diamonds) modeled wet deposition compare to the NTN measurements (black circles).

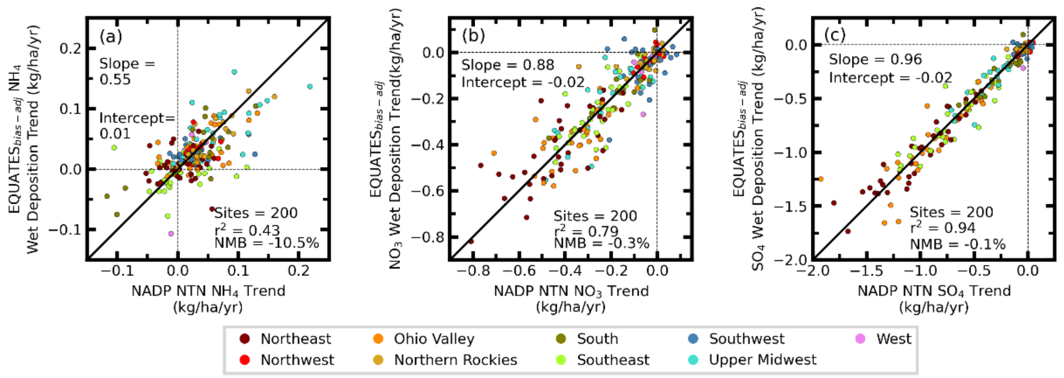

Figure S6. Scatter plot comparing the annual accumulated wet deposition trend (kg/ha/yr) from 2002 to 2017 between NTN observations and EQUATES model output for NH_4 (a), NO_3 (b), and SO_4 (c). Each circle denotes a single NTN site, colored by the climate region that meet annual completeness criteria described in the text.

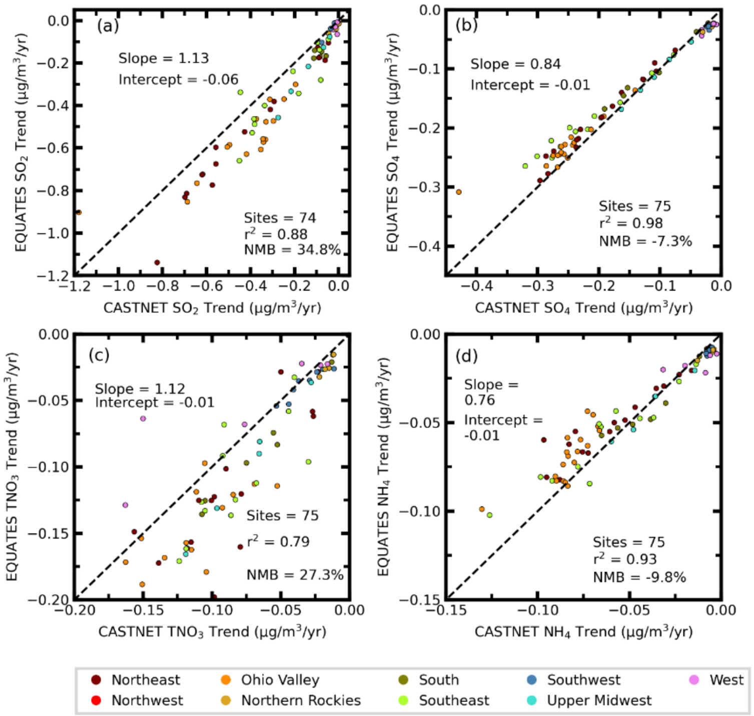

Figure S7. Scatter plot comparing the annual average concentration trend (μg/m^3/yr) from 2002 to 2017 between CASTNET observations and EQUATES model output of sulfur dioxide (a, SO_2), sulfate (b, SO_4), total oxidized nitrogen (c, TNO_3), and ammonium (d, NH_4). Each circle denotes a single CASTNET site colored by the climate that meet annual completeness criteria.

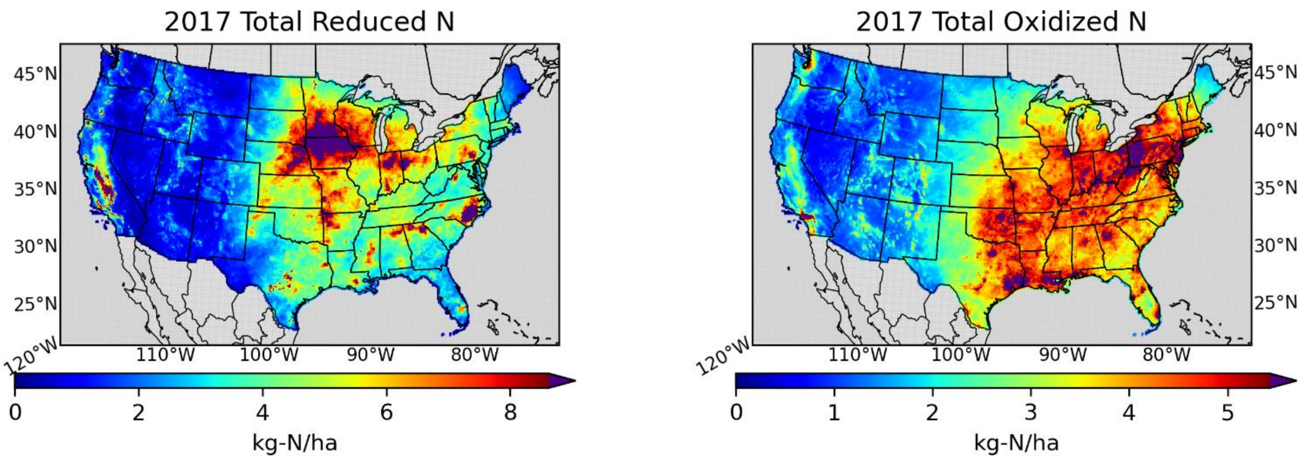

Figure S8. Spatial distribution of 2017 total reduced N deposition (left) and total oxidized N deposition (right). Units are kg-N/ha.

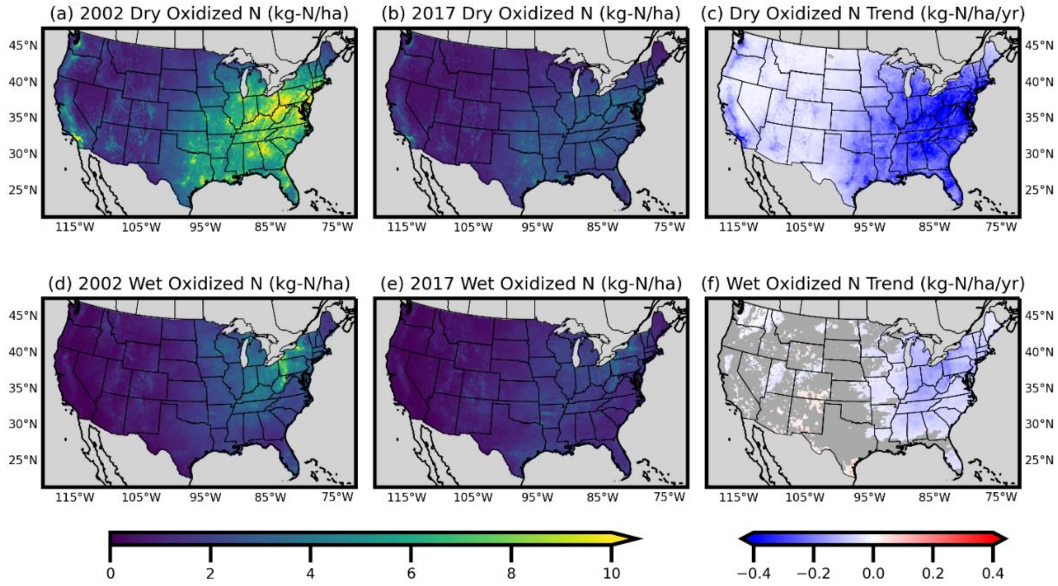

Figure S9. Spatial distribution of dry (top) and wet (bottom) oxidized N deposition in 2002 (a and d, kg-N/ha), 2017 (b and e, kg-N/ha), and the 2002-2017 annual trend (c and f, kg-N/ha/yr) with significance at the 95% confidence interval. Grey areas in panels (c) and (f) indicate where the trend is unavailable or not significant (i.e., p-value of the Wald's test is greater than 0.05).

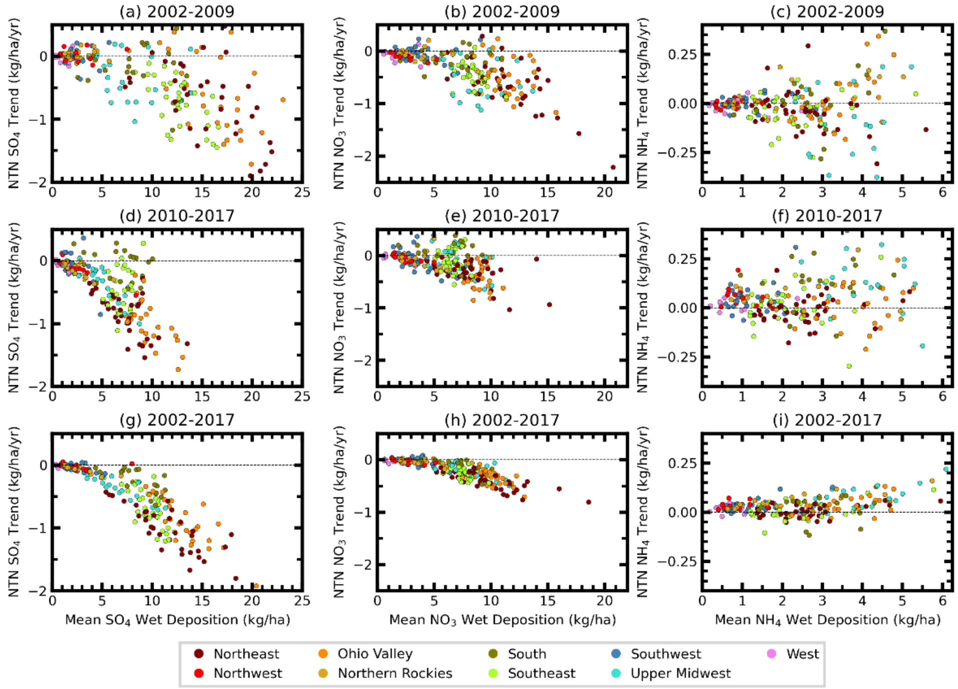

Figure S10. Measured wet deposition trends (kg/ha/yr) of SO_4 (left), NO_3 (middle), and NH_4 (right) as a function of average measured deposition amount from 2002-2009 (top), 2010-2017 (middle), and 2002-2017 (bottom). Each circle is a NTN site colored by the climate region.

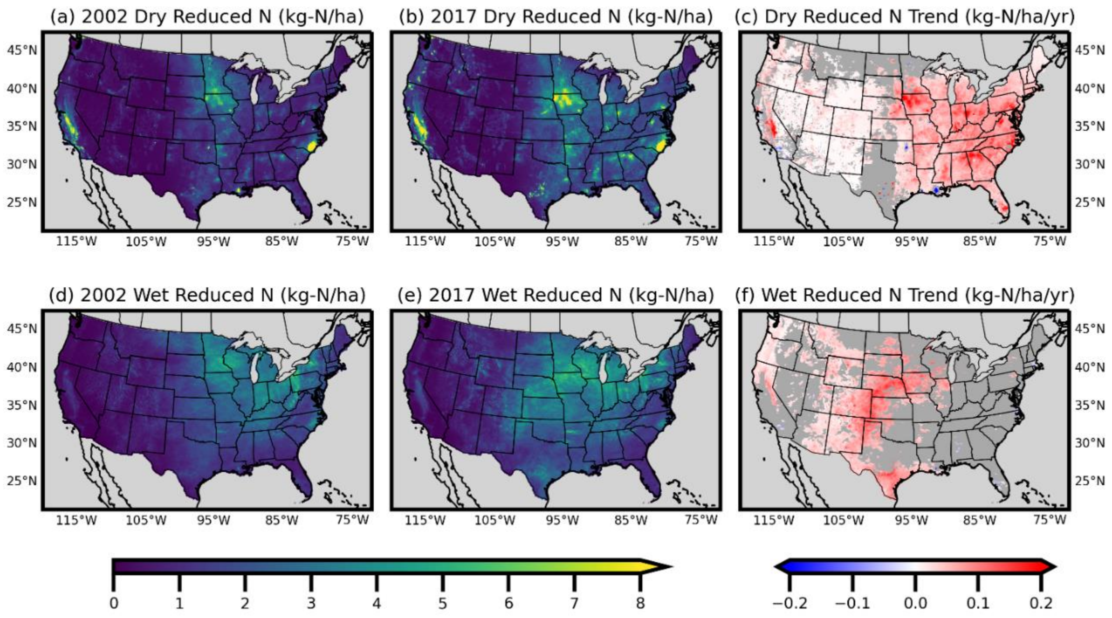

Figure S11. Spatial distribution of dry (top) and wet (bottom) reduced N deposition in 2002 (a and d, kg-N/ha), 2017 (b and e, kg-N/ha), and the 2002-2017 annual trend (c and f, kg-N/ha/yr) with significance at the 95% confidence interval. Grey areas in panels (c) and (f) indicate where the trend is unavailable or not significant (i.e., p-value of the Wald's test is greater than 0.05).

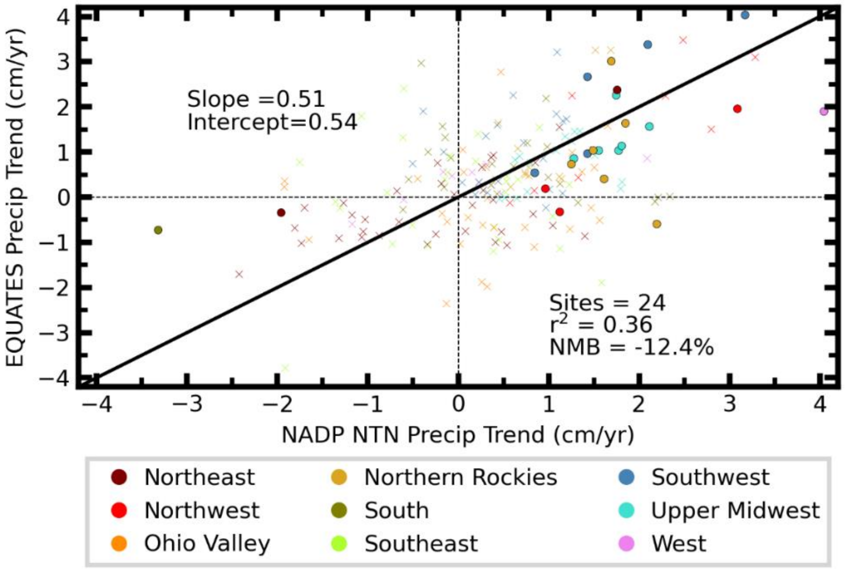

Figure S12. Scatter plot comparing the annual accumulated precipitation trend (cm/yr) from 2002 to 2017 between NTN observations and EQUATES model output. Each marker denotes a single NTN site colored by the climate region that meet annual completeness criteria. Circle markers indicate the observed trend is statistically significant with 95% confidence, while the x's denote unsignificant trends. Summary statistics printed on the figure are computed for sites with statistically significant trends.

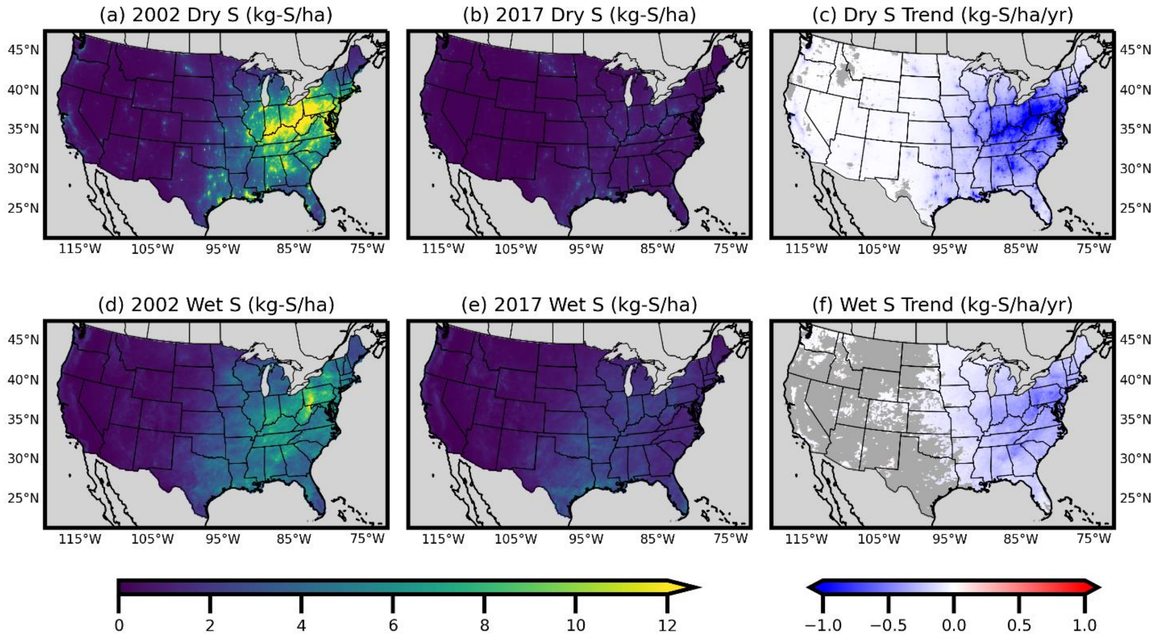

Figure S13. Spatial distribution of dry (top) and wet (bottom) S deposition in 2002 (a and d, kg-S/ha), 2017 (b and e, kg-S/ha), and the 2002-2017 annual trend (c and f, kg-S/ha/yr) with significance at the 95% confidence interval. Grey areas in panels (c) and (f) indicate where the trend is unavailable or not significant (i.e., p-value of the Wald's test is greater than 0.05).

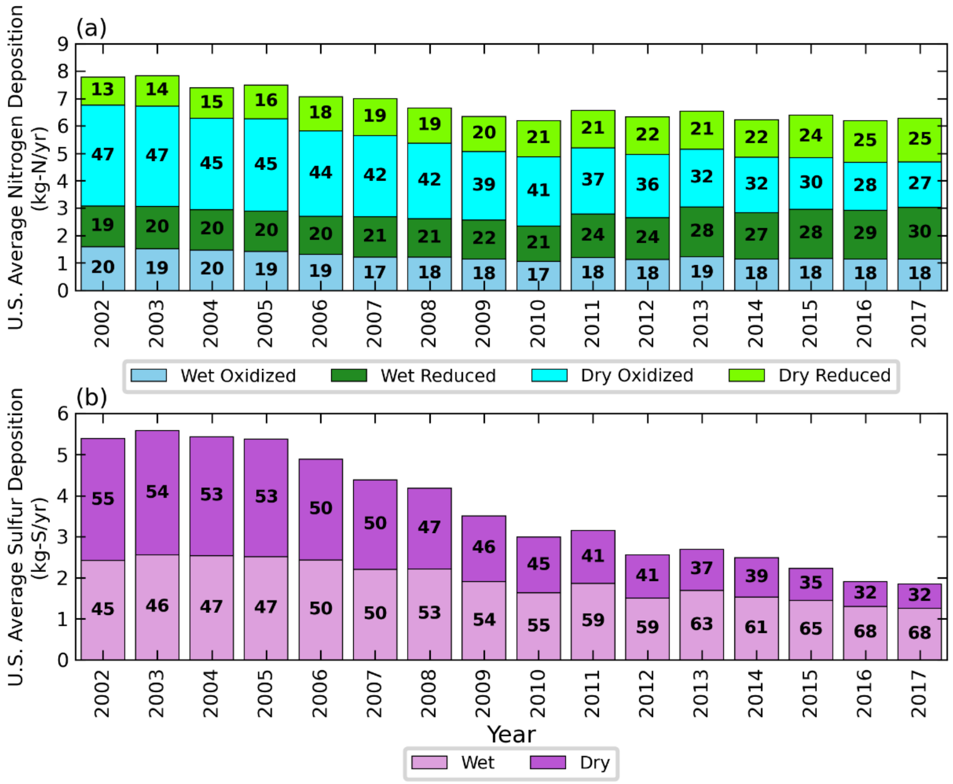

Figure S14. Annual average total (wet+dry) nitrogen (panel a, kg-N/ha) and sulfur (panel b, kg-S/ha) deposition from 2002 to 2017 across the CONUS. The bold numbers in each bar denote the percentage contribution to the annual total.

Table S1. List of NTN site locations assessed in this study with years of valid data, climate region designations, and site class (I=isolated, R=rural, S=suburban, U=urban).

Site ID	Site Name	Latitude	Longitude	Valid Years	NOAA Region	Site Class
AL10	Black Belt Research and Extension Center	32.4583	-87.2422	15	Southeast	I
AL99	Sand Mountain Research and Extension Center	34.2886	-85.9699	16	Southeast	R
AR02	Warren 2WSW	33.605	-92.0972	16	South	R
AR03	Caddo Valley	34.1795	-93.0992	15	South	R
AR16	Buffalo National River-Buffalo Point	36.0842	-92.5868	15	South	I
AR27	Fayetteville	36.1011	-94.1737	16	South	S
AZ03	Grand Canyon National Park-Hopi Point	36.0586	-112.184	16	Southwest	I
AZ06	Organ Pipe Cactus National Monument	31.9492	-112.802	16	Southwest	I
AZ97	Petrified Forest National Park-Rainbow Forest	34.8224	-109.893	14	Southwest	I
AZ98	Chiricahua	32.0097	-109.389	16	Southwest	I
AZ99	Oliver Knoll	33.0708	-109.866	14	Southwest	I
CA42	Tanbark Flat	34.2071	-117.762	15	West	S
CA45	Hopland	39.0045	-123.086	15	West	I
CA66	Pinnacles National Park-Bear Valley	36.4834	-121.157	16	West	I
CA67	Joshua Tree National Park-Black Rock	34.0695	-116.389	15	West	R
CA76	Montague	41.7662	-122.48	16	West	R
CA88	Davis	38.5357	-121.776	15	West	S
CA96	Lassen Volcanic National Park-Manzanita Lake	40.539	-121.577	13	West	I
CA99	Yosemite National Park-Hodgdon Meadow	37.7961	-119.858	14	West	I
CO00	Alamosa	37.4421	-105.868	16	Southwest	R
CO01	Las Animas Fish Hatchery	38.1177	-103.316	16	Southwest	I
CO08	Four Mile Park	39.4025	-107.345	16	Southwest	R
CO10	Gothic	38.9561	-106.986	16	Southwest	I
CO19	Rocky Meadows National Park-Beaver Meadows	40.3639	-105.581	16	Southwest	R
CO21	Manitou	39.1008	-105.093	16	Southwest	R
CO22	Pawnee	40.806	-104.756	14	Southwest	I
CO91	Wolf Creek Pass	37.4686	-106.787	15	Southwest	I
CO92	Sunlight Peak	39.4264	-107.38	15	Southwest	R
CO93	Buffalo Pass-Dry Lake	40.5347	-106.781	16	Southwest	R
CO94	Sugarloaf	39.9939	-105.48	16	Southwest	R
CO96	Molas Pass	37.75	-107.689	15	Southwest	I

CO97	Buffalo Pass-Summit Lake	40.5383	-106.677	16	Southwest	R
CO98	Rocky Mountain National Park-Loch Vale	40.2878	-105.663	13	Southwest	I
CO99	Mesa Verde National Park-Chapin Mesa	37.1979	-108.491	16	Southwest	I
CT15	Abington	41.84	-72.0101	16	Northeast	R
FL03	Bradford Forest	29.9748	-82.1978	14	Southeast	R
FL05	Chassahowitzka National Wildlife Refuge	28.7486	-82.5551	16	Southeast	R
FL11	Everglades National Park-Research Center	25.39	-80.68	16	Southeast	I
FL14	Quincy	30.5486	-84.6004	16	Southeast	R
FL23	Sumatra	30.1106	-84.9902	16	Southeast	I
FL41	Verna Well Field	27.3801	-82.2831	16	Southeast	R
FL99	Kennedy Space Center	28.5428	-80.644	13	Southeast	R
GA09	Okefenokee National Wildlife Refuge	30.7404	-82.1283	16	Southeast	I
GA20	Bellville	32.0849	-81.9367	15	Southeast	R
GA33	Sapelo Island	31.3961	-81.2811	13	Southeast	I
GA41	Georgia Station	33.1805	-84.4103	15	Southeast	R
GA99	Chula	31.5217	-83.5482	16	Southeast	R
IA08	Big Springs Fish Hatchery	42.9097	-91.47	15	Upper Midwest	I
IA23	McNay Research Center	40.9631	-93.3925	16	Upper Midwest	R
ID02	Priest River Experimental Forest	48.3518	-116.84	15	Northwest	I
ID03	Craters of the Moon National Monument	43.4605	-113.555	16	Northwest	I
ID11	Reynolds Creek	43.2049	-116.75	16	Northwest	I
IL11	Bondville	40.0528	-88.3719	16	Ohio Valley	S
IL18	Shabbona	41.8414	-88.8511	14	Ohio Valley	R
IL46	Alhambra	38.8689	-89.6219	16	Ohio Valley	R
IL63	Dixon Springs Agricultural Center	37.4356	-88.6719	15	Ohio Valley	I
IL78	Monmouth	40.9333	-90.7231	16	Ohio Valley	R
IN20	Roush Lake	40.8401	-85.4639	16	Ohio Valley	R
IN22	Southwest Purdue Agriculture Center	38.7408	-87.4855	15	Ohio Valley	R
IN34	Indiana Dunes National Lakeshore	41.6318	-87.0881	16	Ohio Valley	S
IN41	Agronomy Center for Research and Extension	40.4749	-86.9924	16	Ohio Valley	S
KS07	Farlington Fish Hatchery	37.6511	-94.8036	16	South	I
KS31	Konza Prairie	39.1022	-96.6092	16	South	R
KS32	Lake Scott State Park	38.6717	-100.916	16	South	I
KY03	Mackville	37.7047	-85.0489	16	Ohio Valley	R
KY10	Mammoth Cave National Park-Houchin Meadow	37.1317	-86.148	15	Ohio Valley	R

KY19	Cannons Lane	38.2288	-85.6545	13	Ohio Valley	U
KY22	Lilley Cornett Woods	37.0778	-82.9936	16	Ohio Valley	R
KY35	Clark State Fish Hatchery	38.1183	-83.5469	14	Ohio Valley	R
KY99	Mulberry Flatt	36.9029	-88.0121	16	Ohio Valley	I
LA30	Southeast Research Station	30.7819	-90.2021	16	South	R
MA01	North Atlantic Coastal Lab	41.9759	-70.0241	15	Northeast	R
MA08	Quabbin Reservoir	42.3925	-72.3444	16	Northeast	R
MD13	Wye	38.9131	-76.1525	16	Northeast	R
MD15	Smith Island	37.9925	-76.0345	13	Northeast	I
MD18	Assateague Island National Seashore-Woodcock	38.251	-75.1593	16	Northeast	R
MD99	Beltsville	39.028	-76.8171	13	Northeast	U
ME00	Caribou	46.8675	-68.0134	15	Northeast	R
ME02	Bridgton	44.1075	-70.7289	16	Northeast	R
ME04	Carrabassett Valley	45.0803	-70.2118	13	Northeast	I
ME09	Greenville Station	45.4891	-69.6647	15	Northeast	I
ME96	Casco Bay-Wolfe’s Neck Farm	43.8325	-70.0645	16	Northeast	R
ME98	Acadia National Park-McFarland Hill	44.3772	-68.2608	16	Northeast	R
MI09	Douglas Lake	45.5608	-84.6783	16	Upper Midwest	R
MI26	Kellogg Biological Station	42.4103	-85.3928	16	Upper Midwest	R
MI48	Seney National Wildlife Refuge-Headquarters	46.2889	-85.9504	16	Upper Midwest	I
MI51	Unionville	43.6135	-83.3599	16	Upper Midwest	R
MI52	Ann Arbor	42.4164	-83.9019	16	Upper Midwest	S
MI53	Wellston	44.2242	-85.8186	16	Upper Midwest	I
MI99	Chassell	47.1046	-88.5516	16	Upper Midwest	R
MN01	Cedar Creek	45.4017	-93.2031	16	Upper Midwest	R
MN08	Hovland	47.8471	-89.965	16	Upper Midwest	I
MN16	Marcell Experimental Forest	47.5311	-93.4686	16	Upper Midwest	I
MN18	Fernberg	47.9464	-91.4961	15	Upper Midwest	I
MN23	Camp Ripley	46.2494	-94.4972	15	Upper Midwest	I
MN27	Lamberton	44.237	-95.3011	15	Upper Midwest	I
MN28	Grindstone Lake	46.1217	-93.0001	16	Upper Midwest	R
MN32	Voyageurs National Park-Sullivan Bay	48.4132	-92.8305	15	Upper Midwest	I
MN99	Wolf Ridge	47.3841	-91.2067	16	Upper Midwest	I
MO03	Ashland Wildlife Area	38.754	-92.1994	15	Ohio Valley	R
MO05	University Forest	36.9108	-90.3187	16	Ohio Valley	R

MS10	Clinton	32.3069	-90.3186	14	South	S
MS19	Newton	32.3269	-89.2086	15	South	R
MS30	Coffeeville	34.0025	-89.7993	13	South	I
MT00	Little Bighorn Battlefield National Monument	45.5701	-107.438	14	Northern Rockies	I
MT05	Glacier National Park-Fire Weather Station	48.5102	-113.997	16	Northern Rockies	I
MT07	Clancy	46.485	-112.065	14	Northern Rockies	R
MT98	Havre-Northern Agricultural Research Center	48.5007	-109.798	15	Northern Rockies	R
NC03	Lewiston	36.1325	-77.1708	16	Southeast	I
NC06	Beaufort	34.8846	-76.6207	15	Southeast	R
NC25	Coweeta	35.0605	-83.4305	16	Southeast	R
NC29	Hofmann Forest	34.825	-77.3228	15	Southeast	S
NC34	Piedmont Research Station	35.697	-80.6225	16	Southeast	R
NC35	Clinton Crops Research Station	35.0258	-78.2783	16	Southeast	R
NC36	Jordan Creek	34.9705	-79.5281	15	Southeast	R
NC41	Finley Farm	35.7288	-78.6802	16	Southeast	U
ND00	Theodore Roosevelt National Park-Painted Canyon	46.8951	-103.378	16	Northern Rockies	I
ND08	Icelandic State Park	48.782	-97.7546	13	Northern Rockies	I
ND11	Woodworth	47.1247	-99.2381	15	Northern Rockies	I
NE15	Mead	41.1528	-96.4912	16	Northern Rockies	R
NE99	North Platte Agricultural Experiment Station	41.0592	-100.746	14	Northern Rockies	R
NH02	Hubbard Brook	43.9433	-71.7029	16	Northeast	R
NJ00	Edwin B. Forsythe National Wildlife Refuge	39.4728	-74.4369	15	Northeast	S
NJ99	Washington Crossing	40.3154	-74.8536	16	Northeast	U
NM07	Bandelier National Monument	35.7788	-106.266	16	Southwest	R
NM08	Mayhill	32.9096	-105.471	14	Southwest	I
NV03	Smith Valley	38.7992	-119.257	16	West	I
NV05	Great Basin National Park-Lehman Caves	39.0054	-114.217	15	West	I
NY01	Alfred	42.2276	-77.8016	13	Northeast	R
NY08	Aurora Research Farm	42.7339	-76.6597	16	Northeast	R
NY10	Chautauqua	42.2994	-79.3964	15	Northeast	R
NY20	Huntington Wildlife	43.9731	-74.2231	16	Northeast	I
NY22	Akwesasne Mohawk-Fort Covington	44.9226	-74.4806	15	Northeast	R
NY52	Bennett Bridge	43.5282	-75.9492	13	Northeast	R
NY68	Biscuit Brook	41.9936	-74.5031	16	Northeast	I
NY96	Cedar Beach-Southold	41.0347	-72.3891	13	Northeast	R

NY98	Whiteface Mountain	44.3933	-73.8594	16	Northeast	I
NY99	West Point	41.3511	-74.0484	16	Northeast	S
OH09	Oxford	39.5309	-84.7238	16	Ohio Valley	R
OH17	Delaware	40.3555	-83.0661	16	Ohio Valley	R
OH49	Caldwell	39.7928	-81.5311	16	Ohio Valley	R
OH54	Deer Creek State Park	39.6359	-83.2606	16	Ohio Valley	R
OH71	Wooster	40.7813	-81.9197	16	Ohio Valley	R
OK00	Salt Northern Rockies National Wildlife Refuge	36.7863	-98.18	16	South	I
OK17	Kessler Farm Field Laboratory	34.98	-97.5214	15	South	R
OK29	Goodwell Research Station	36.5908	-101.618	16	South	I
OR10	H. J. Andrews Experimental Forest	44.2118	-122.256	16	Northwest	I
OR18	Starkey Experimental Forest	45.2247	-118.513	16	Northwest	I
OR97	Hyslop Farm	44.6347	-123.19	16	Northwest	S
PA00	Arendtsville	39.9231	-77.3078	16	Northeast	R
PA15	Penn State	40.7883	-77.9458	16	Northeast	S
PA18	Young Woman’s Creek	41.4142	-77.6799	16	Northeast	I
PA29	Kane Experimental Forest	41.5978	-78.7675	16	Northeast	R
PA42	Leading Ridge	40.6575	-77.9397	16	Northeast	R
PA47	Millersville	39.9909	-76.3856	14	Northeast	S
PA72	Milford	41.3273	-74.8199	13	Northeast	R
SC05	Cape Romain National Wildlife Refuge	32.943	-79.6592	16	Southeast	R
SC06	Santee National Wildlife Refuge	33.5394	-80.435	15	Southeast	R
SD04	Wind Cave National Park-Elk Mountain	43.5577	-103.484	14	Northern Rockies	I
SD08	Cottonwood	43.9461	-101.855	15	Northern Rockies	I
SD99	Huron Well Field	44.355	-98.2917	16	Northern Rockies	R
TN04	Speedwell	36.4698	-83.8272	16	Ohio Valley	R
TN11	Great Smoky Mountains National Park-Elkmount	35.6645	-83.5903	16	Ohio Valley	R
TN14	Hatchie National Wildlife Refuge	35.4688	-89.1713	14	Ohio Valley	I
TX03	Beeville	28.4667	-97.7069	16	South	R
TX04	Big Bend National Park-K-Bar	29.3025	-103.178	15	South	I
TX10	Attwater Prairie Chicken National Wildlife Refuge	29.6614	-96.2594	14	South	R
TX16	Sonora	30.2613	-100.555	16	South	I
TX21	Longview	32.3786	-94.7117	16	South	R
TX22	Guadalupe Mountains National Park Frijole Ranger Station	31.9069	-104.805	15	South	I
TX56	L.B.J. National Grasslands	33.3917	-97.6397	16	South	I

UT01	Logan	41.6661	-111.891	15	Southwest	S
UT09	Canyonlands National Park-Island in the Sky	38.4584	-109.821	15	Southwest	I
UT98	Green River	39.0001	-110.174	16	Southwest	I
UT99	Bryce Canyon National Park-Repeater Hill	37.6186	-112.173	16	Southwest	I
VA00	Charlottesville	38.0402	-78.5427	16	Southeast	S
VA13	Hortons Station	37.3232	-80.4572	16	Southeast	R
VA24	Prince Edward	37.1652	-78.3073	16	Southeast	R
VA28	Shenandoah National Park-Big Meadows	38.5231	-78.4348	15	Southeast	R
VA99	Natural Bridge Station	37.6265	-79.5126	15	Southeast	R
VT01	Bennington	42.8761	-73.1633	16	Northeast	R
VT99	Underhill	44.5283	-72.8684	16	Northeast	R
WA14	Olympic National Park-Hoh Ranger Station	47.8597	-123.933	14	Northwest	I
WA19	North Cascades National Park-Marblemount Ranger Station	48.5403	-121.446	16	Northwest	I
WA21	La Grande	46.8353	-122.287	14	Northwest	R
WA24	Palouse Conservation Farm	46.7606	-117.185	16	Northwest	R
WA98	Columbia River Gorge	45.5694	-122.21	14	Northwest	R
WA99	Mount Rainier National Park-Tahoma Woods	46.7582	-122.124	15	Northwest	I
WI35	Perkinstown	45.2064	-90.5978	16	Upper Midwest	I
WI36	Trout Lake	46.0512	-89.6541	16	Upper Midwest	I
WI37	Spooner	45.8228	-91.8744	15	Upper Midwest	R
WV05	Cedar Creek State Park	38.8794	-80.8476	15	Ohio Valley	R
WV18	Parsons	39.0897	-79.6622	16	Ohio Valley	I
WY00	Snowy Range	41.3762	-106.26	16	Northern Rockies	I
WY02	Sinks Canyon	42.7336	-108.85	16	Northern Rockies	I
WY06	Pinedale	42.929	-109.788	13	Northern Rockies	I
WY08	Yellowstone National Park-Tower Falls	44.9166	-110.42	16	Northern Rockies	I
WY95	Brooklyn Lake	41.3647	-106.241	16	Northern Rockies	I
WY98	Gypsum Creek	43.2227	-109.992	13	Northern Rockies	I
WY99	Newcastle	43.873	-104.192	16	Northern Rockies	I

Table S2. Location of CASTNET sites assessed in this study with years of valid data and climate region designations.

Site ID	Latitude	Longitude	NOAA Region	Valid Years
ABT147	41.8405	-72.0104	Northeast	16
ACA416	44.3771	-68.2608	Northeast	16
ALC188	30.7016	-94.674	South	14
ALH157	38.869	-89.6228	Ohio Valley	16
ANA115	42.4166	-83.9022	Upper Midwest	16
ARE128	39.9232	-77.3079	Northeast	16
ASH135	46.6038	-68.4132	Northeast	16
BBE401	29.3027	-103.178	South	16
BEL116	39.0282	-76.8171	Northeast	15
BFT142	34.8847	-76.6207	Southeast	16
BVL130	40.052	-88.3725	Ohio Valley	16
BWR139	38.445	-76.1113	Northeast	16
CAD150	34.1793	-93.0988	South	16
CAN407	38.4583	-109.821	Southwest	16
CAT175	41.9423	-74.552	Northeast	16
CDR119	38.8795	-80.8477	Ohio Valley	16
CDZ171	36.7841	-87.8502	Ohio Valley	16
CHA467	32.0094	-109.389	Southwest	16
CHE185	35.7508	-94.6698	South	16
CKT136	37.9215	-83.0663	Ohio Valley	16
CND125	35.2633	-79.8375	Southeast	16
CNT169	41.3645	-106.24	Northern Rockies	16
COW137	35.0605	-83.4303	Southeast	16
CTH110	42.4009	-76.6535	Northeast	16
CVL151	34.0027	-89.7992	South	16
DCP114	39.6359	-83.2606	Ohio Valley	16
ESP127	36.0389	-85.733	Ohio Valley	16
EVE419	25.3912	-80.6808	Southeast	15
GAS153	33.1812	-84.4101	Southeast	16
GLR468	48.5103	-113.997	Northern Rockies	16
GRB411	39.0051	-114.216	West	15
GRC474	36.0586	-112.184	Southwest	16
GRS420	35.6335	-83.9416	Ohio Valley	15
GTH161	38.9563	-106.986	Southwest	16
HOX148	44.1809	-85.739	Upper Midwest	15
HWF187	43.973	-74.2233	Northeast	16
IRL141	27.8492	-80.4556	Southeast	15
JOT403	34.0696	-116.389	West	16
KEF112	41.5981	-78.7679	Northeast	15
KNZ184	39.1022	-96.6096	South	16

LAV410	40.54	-121.577	West	16
LRL117	39.9883	-79.2516	Northeast	16
MAC426	37.1318	-86.143	Ohio Valley	16
MCK131	37.7047	-85.0487	Ohio Valley	15
MCK231	37.7047	-85.0487	Ohio Valley	16
MEV405	37.1984	-108.491	Southwest	14
MKG113	41.4268	-80.1452	Northeast	16
OXF122	39.5311	-84.7235	Ohio Valley	16
PAR107	39.0904	-79.6617	Ohio Valley	16
PED108	37.1652	-78.3071	Southeast	13
PET427	34.8225	-109.893	Southwest	16
PIN414	36.4832	-121.157	West	16
PND165	42.929	-109.788	Northern Rockies	16
PNF126	36.1054	-82.045	Southeast	16
PRK134	45.2065	-90.5972	Upper Midwest	15
PSU106	40.7209	-77.9318	Northeast	16
QAK172	39.9427	-81.3379	Ohio Valley	16
ROM206	40.2781	-105.546	Southwest	16
ROM406	40.2781	-105.546	Southwest	16
SAL133	40.816	-85.6614	Ohio Valley	15
SEK430	36.4895	-118.829	West	13
SHN418	38.5231	-78.4347	Southeast	15
SND152	34.289	-85.9701	Southeast	15
SPD111	36.4698	-83.8265	Ohio Valley	15
STK138	42.2872	-89.9999	Ohio Valley	16
SUM156	30.1102	-84.9904	Southeast	16
THR422	46.8948	-103.378	Northern Rockies	15
UVL124	43.6136	-83.3599	Upper Midwest	16
VIN140	38.7408	-87.4849	Ohio Valley	16
VOY413	48.4125	-92.8292	Upper Midwest	16
VPI120	37.3232	-80.4572	Southeast	16
WSP144	40.3123	-74.8727	Northeast	16
WST109	43.9445	-71.7008	Northeast	15
YEL408	44.5654	-110.4	Northern Rockies	16
YOS404	37.7132	-119.706	West	16

Table S3. Uncorrected EQUATES model performance metrics of seasonal and annual accumulated wet deposition of NO_3, NH_4, SO_4, and precipitation.

		Winter	Spring	Summer	Fall	Annual, no adjustment (Table 1)
NO_3	r^2	0.63	0.74	0.60	0.69	0.77
	MB (kg/ha)	0.01	0.09	0.04	0.23	0.55
	NMB (%)	1.37	5.44	2.22	20.90	9.64
NH_4	r^2	0.50	0.63	0.49	0.55	0.61
	MB (kg/ha)	-0.10	-0.23	-0.03	-0.10	-0.49
	NMB (%)	-39.7	-34.8	-3.70	-25.0	-19.9
SO_4	r^2	0.65	0.71	0.72	0.67	0.78
	MB (kg/ha)	-0.17	-0.26	-0.30	-0.17	-0.92
	NMB (%)	-18.9	-14.3	-14.5	-12.9	-12.2
Precipitation	r^2	0.77	0.70	0.61	0.69	0.72
	MB (kg/ha)	3.63	-2.66	-12.9	-23.2	-2.32
	NMB (%)	2.28	-1.30	-5.88	-11.7	-2.40%
